# Phenotypic and genomic hallmarks of a novel, potentially pathogenic rapidly growing *Mycobacterium* species related to the *Mycobacterium fortuitum* complex

**DOI:** 10.1038/s41598-021-91737-8

**Published:** 2021-06-21

**Authors:** Reem Gharbi, Varun Khanna, Wafa Frigui, Besma Mhenni, Roland Brosch, Helmi Mardassi

**Affiliations:** 1 Unit of Typing & Genetics of Mycobacteria, Laboratory of Molecular Microbiology, Vaccinology, and Biotechnology Development, Institut Pasteur de Tunis, Université de Tunis El Manar, Tunis, Tunisia; 2 Institut Pasteur, Hub Bioinformatique et Biostatistique, C3BI, Unité de Services et de Recherche, USR 3756, Institut Pasteur CNRS, Paris, France; 3 Institut Pasteur (IP), Unit for Integrated Mycobacterial Pathogenomics, 75015 Paris, France

**Keywords:** Genetics, Microbiology

## Abstract

Previously, we have identified a putative novel rapidly growing *Mycobacterium* species, referred to as TNTM28, recovered from the sputum of an apparently immunocompetent young man with an underlying pulmonary disease. Here we provide a thorough characterization of TNTM28 genome sequence, which consists of one chromosome of 5,526,191 bp with a 67.3% G + C content, and a total of 5193 predicted coding sequences. Phylogenomic analyses revealed a deep-rooting relationship to the *Mycobacterium fortuitum* complex, thus suggesting a new taxonomic entity. TNTM28 was predicted to be a human pathogen with a probability of 0.804, reflecting the identification of several virulence factors, including export systems (Sec, Tat, and ESX), a nearly complete set of Mce proteins, toxin-antitoxins systems, and an extended range of other genes involved in intramacrophage replication and persistence (hspX, ahpC, sodA, sodC, katG, mgtC, ClpR, virS, etc.), some of which had likely been acquired through horizontal gene transfer. Such an arsenal of potential virulence factors, along with an almost intact ESX-1 locus, might have significantly contributed to TNTM28 pathogenicity, as witnessed by its ability to replicate efficiently in macrophages. Overall, the identification of this new species as a potential human pathogen will help to broaden our understanding of mycobacterial pathogenesis.

## Introduction

Non tuberculous mycobacteria (NTM) are environmental germs that can invade a host and cause lung, skin or lymphatic infections in immunocompetent patients or cause disseminated infections, particularly in immunocompromised individuals^1–3^. Pulmonary infections due to NTM are increasingly recognized worldwide through improved culture and identification techniques^4,5^. NTM species are generally subdivided on the basis of growth into rapid and slow growing mycobacteria (RGM and SGM, respectively)^6^. Like the pathogenic *Mycobacterium tuberculosis* complex (MTBC) members, pathogenicity in NTM is predominantly correlated with slow growth^7^, yet some RGM species have been associated with true microbiological diseases^8^.

Among RGM, *Mycobacterium abscessus, Mycobacterium fortuitum, and Mycobacterium chelonae,* account amongst the most clinically relevant specie*s*^8^*. M. abscessus* is the most frequently isolated from clinical respiratory specimens, while *M. fortuitum* is the most common from non-respiratory specimens. The latter species, mainly found in soil, dust, water, and animal sources, encompasses a large group of emerging opportunistic pathogens, generally subdivided into three biovars, referred to as the *M. fortuitum complex*^9–11^. Reports on novel *M. fortuitum*-related species have significantly increased over the past decades^12,13^. Currently, the *M. fortuitum* complex consists of a monophyletic group of several species including, but not limited to, *M. fortuitum* stricto sensus (strain CT6), *Mycobacterium mageritense, Mycobacterium conceptionense, Mycobacterium septicum*, *Mycobacterium peregrinum*, *Mycobacterium porcinum*, and *Mycobacterium senegalense*^14^.

Infection with *M. fortuitum* complex species can cause a variety of clinical diseases, being most frequently associated with skin and soft tissues in both immunocompetent and immunocompromised patients^15,16^. The presence of *M. fortuitum* species in the respiratory tract has been mainly reported following simple colonization or ephemeral infection. However, a true lung infection due to *M. fortuitum* remains infrequent, and generally occurs in patients with gastroesophageal disease or in elderly patients with chronic cough^17–19^. Isolation of new *M. fortuitum* complex species, particularly those associated with pulmonary diseases, is thus worthy of consideration since it could provide new clues to better understand the evolution and pathogenesis of this mycobacterial group.

Previously, we have described a putative new RGM species, referred to as TNTM28, isolated from the sputum of an apparently immunocompetent young man presenting with an underlying pulmonary disease^20^. Based on sequence polymoprphisms in 16 rRNA, *hsp65*, and *rpoB* gene sequences, this non photochromogenic RGM was found to be related to the *M. fortuitum* complex. Here we provide a genome-based description of TNTM28, which is confirming its phylogenetic link to the *M. fortuitum* complex, but also shows enough differences to justify the status of a new species. TNTM28 was found to display several features reminiscent of a pathogenic species.

## Results

### Phenotypic characterization and further genetic analysis of TNTM28

**Figure 1:**
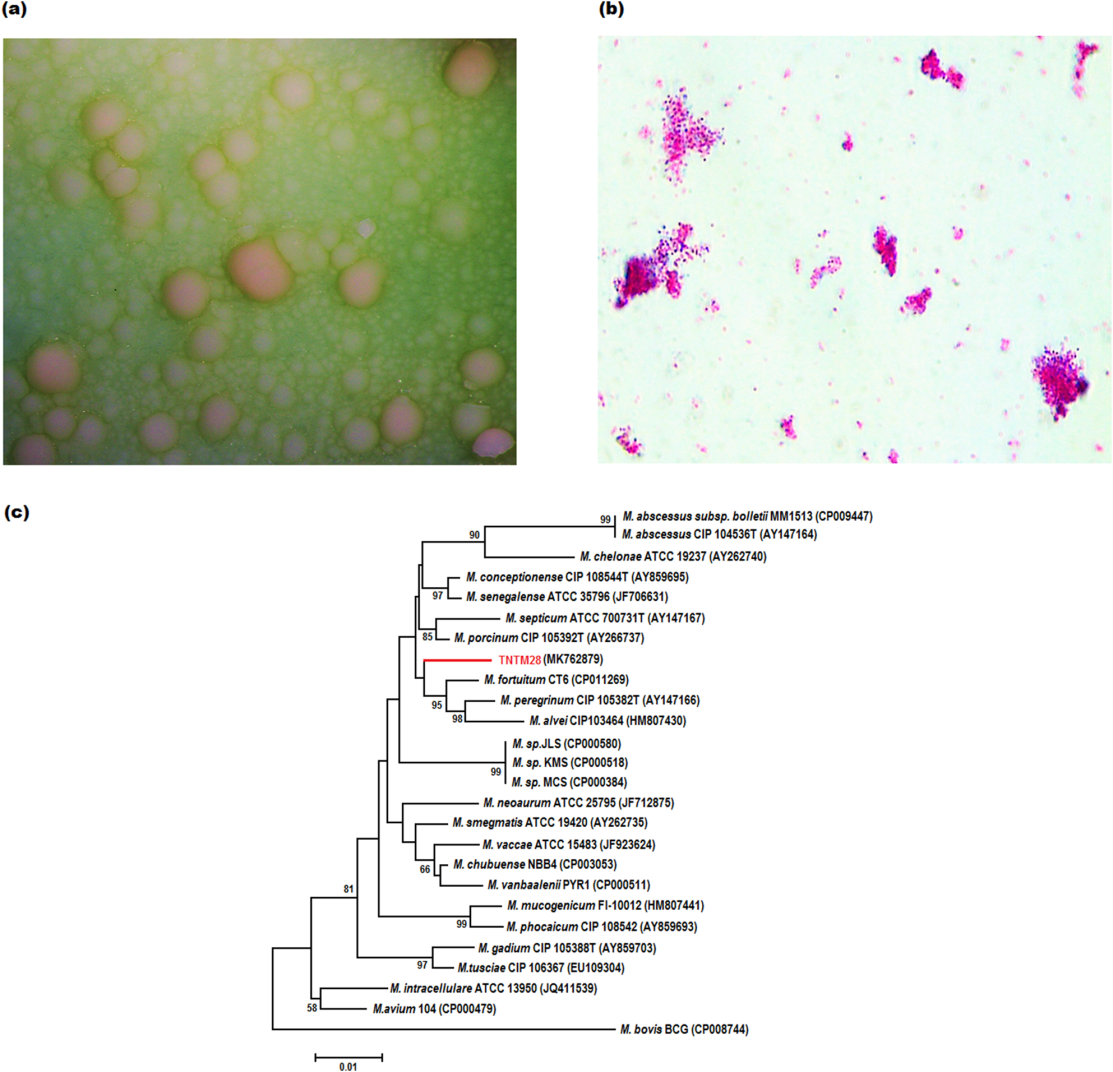
Phenotypic appearance and phylogeny of TNTM28. (**a**) Morphotype of TNTM28 on LJ medium. (**b**) TNTM28 bacilli as observed after Ziehl-Neelsen staining. (**c**) Phylogenetic tree based on *rpoB* gene sequence highlighting the position of strain TNTM28 relative to other NTM strains.

In Löwenstein-Jensen (LJ) medium, TNTM28 appeared as small, nonpigmented, hemishperic colonies (approx. 1 mm in diameter) with a rough morphotype, mostly grouped in rosettes (Fig. 1a). Microscopic analysis of TNTM28 bacilli showed a Gram-positive type, and also displayed red color after Ziehl–Neelsen straining, where the bacteria tended to form large aggregates (Fig. 1b). TNTM28 colonies grew on LJ agar within 2 to 4 days at 37 °C (optimum), in the presence or absence of 5% NaCl. Growth did also occur at 30 °C, albeit less efficiently than at temperatures between 33 and 37 °C. No growth occurred at 42 °C.

Biochemical tests showed that TNTM28 was niacin-negative, but proved positive when tested for arylsulfatase production (after 3 and 14 days), alkaline phosphatase, nitrate reductase, and thermostable catalase activities. TNTM28 was capable to hydrolyse Tween-80 and urea, and was found competent for iron uptake.

As mentioned above, previous analyses based on 16S rRNA and *rpoB* gene sequence polymorphism revealed that TNTM28 was phylogenetically related to members of the *M. fortuitum* complex. Here, we refined such a phylogenetic analysis by including a larger dataset of the *M. fortuitum* complex group. As shown in the *rpoB*-based phylogenetic tree depicted in Fig. 1c, TNTM28 was related to the *M. fortuitum* complex, but proved quite divergent from other species, being deeply rooted to *Mycobacterium septicum* strain ATCC 700731^T^ (AY147167), *Mycobacterium alvei* CIP103464 (HM807430)*,* and *Mycobacterium peregrinum* CIP 105382^T^ (AY147166) with 94.49%, 94.23% and 94.06% *rpoB* gene sequence similarity, respectively.

### General features of TNTM28 genome

**Figure 2:**
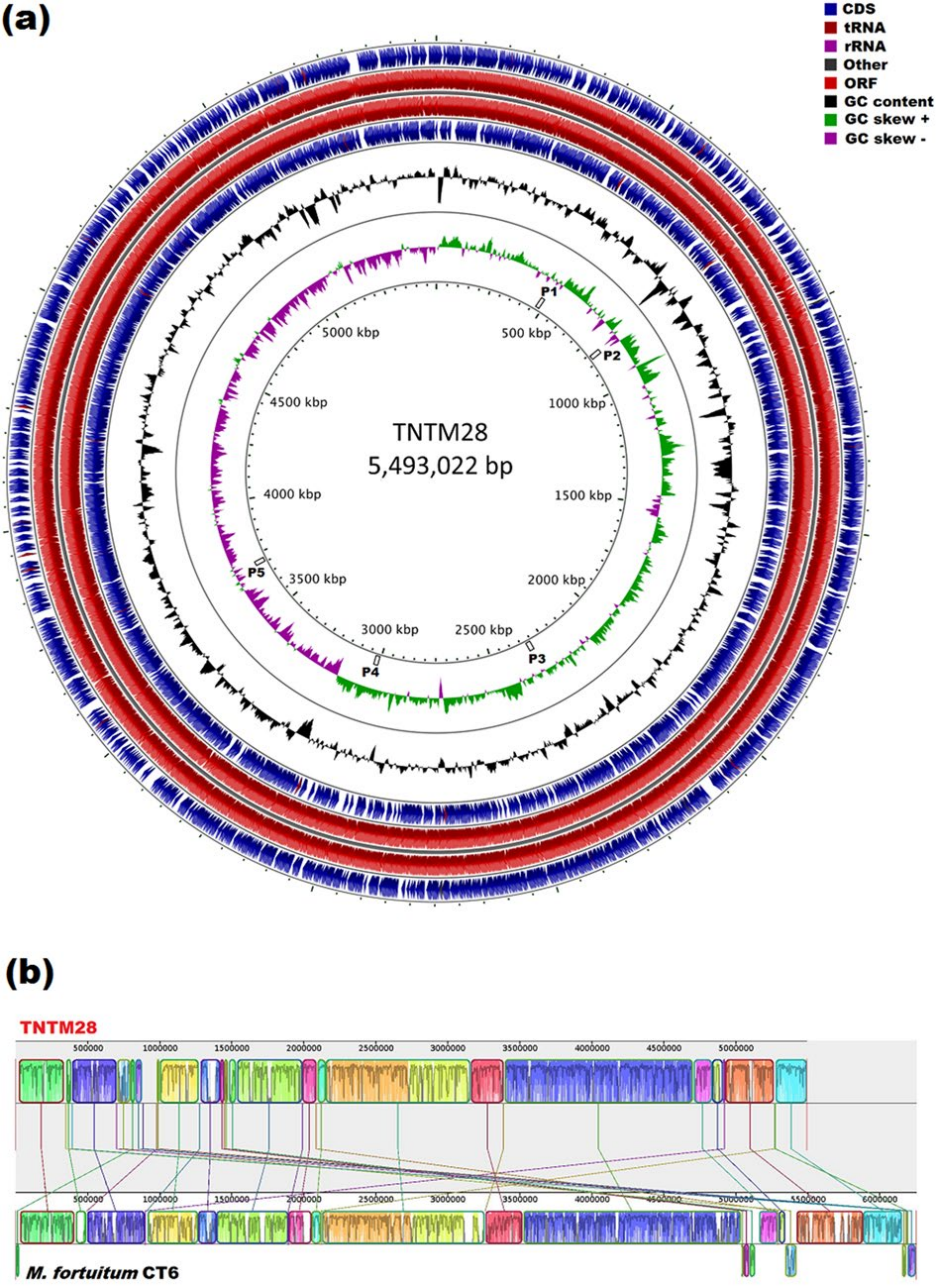
Graphical map of TNTM28 genome. (**a**) Circular map of the 5.49 Mb TNTM28 chromosome performed with GCview Server. The circles represent, from outside to inside: rings 1 and 4 show protein-coding genes oriented in the forward and reverse orientations, respectively. Rings 2 and 3 display genes on forward and reverse strand, respectively. Ring 5 shows G + C% content plot (black). Ring 6 shows GC skews, with positive and negative values being indicated with green and purple colors, respectively. Positions of the prophage regions (P1 to P5) are indicated in the innermost circle. (**b**) Genome alignment performed using Mauve software between TNTM28 with its closest species, *M. foruitum*, strain CT6. Boxes with identical colors represent locally collinear blocks (LCBs), indicating homologous DNA regions shared between the two genomes without sequence rearrangement. Lines collate aligned segments between genomes. The vertical bars denote the conservation level, and upward and downward orientations relative to the genome line indicates collinear and inverted regions, respectively. Sequences outside colored blocks do not have homologs in the other genome. Red lines indicate contig boundaries within the assembly.

After assembling and filtering, based on median coverage ≥ 100 X, the pre-processed 5,469,922 paired-end reads resulted in 50 scaffolds with a total length of 5,526,191 bp. Using the genome of *M. fortuitum* strain CT6 (the nearest genome sequence) as reference, 28 scaffolds (5,493,022 bp) were mapped, while the remaining 22 (35,969 bp) proved unplaced. The maximum length of scaffolds was estimated to be 851,167 bp. The assembly had N50 and N90 values of 338,742 pb and 103,464 pb, respectively. The genome of TNTM28 displayed a high G + C content of 67.3%, and contained no plasmid replicons. A total of 5193 protein-coding genes (CDS) was identifed, along with 52 tRNA, 1 tmRNA, a single 16S-5S-23S ribosomal RNA operon, and 3 putative CRISPRs loci (Table 1). A circular representation of TNTM28 genome is shown in Fig. 2a. The genome sequence of TNTM28 showed no major genomic rearrangements, being mostly syntenic to that of *M. fortuitum* CT6 strain (Fig. 2b). Five incomplete prophage regions (A to E) have been identified. They mainly consisted of hypothetical and phage-like protein sequences (Supplementary Figure S1).

**Table 1:** General features of TNTM28 genome.

Attribute	Genome (total)	
	Value	% of total
Genome (bp)	5,702,200	100
Coding DNA	5,526,191	96.91
G + C (bp)	3,837,580	67.3
Total length of mapped scaffolds (bp)	5,490,222	96.28
Total length of unplaced scaffolds (pb)	35,969	3.78
Total proteins	5266	100
Total protein codin genes (mapped scaffolds)	5193	98.61
Total RNA	54	1.02
Putative proteins	646	12.26
Genes in paralogs clusters	422	8.12
Genes assigned to COGs	4009	76.12
Uniques clusters of proteins	57	1.08
Hypothetical proteins	2078	39.46
CRISPR	3	0.056

### Functional annotation

**Figure 3:**
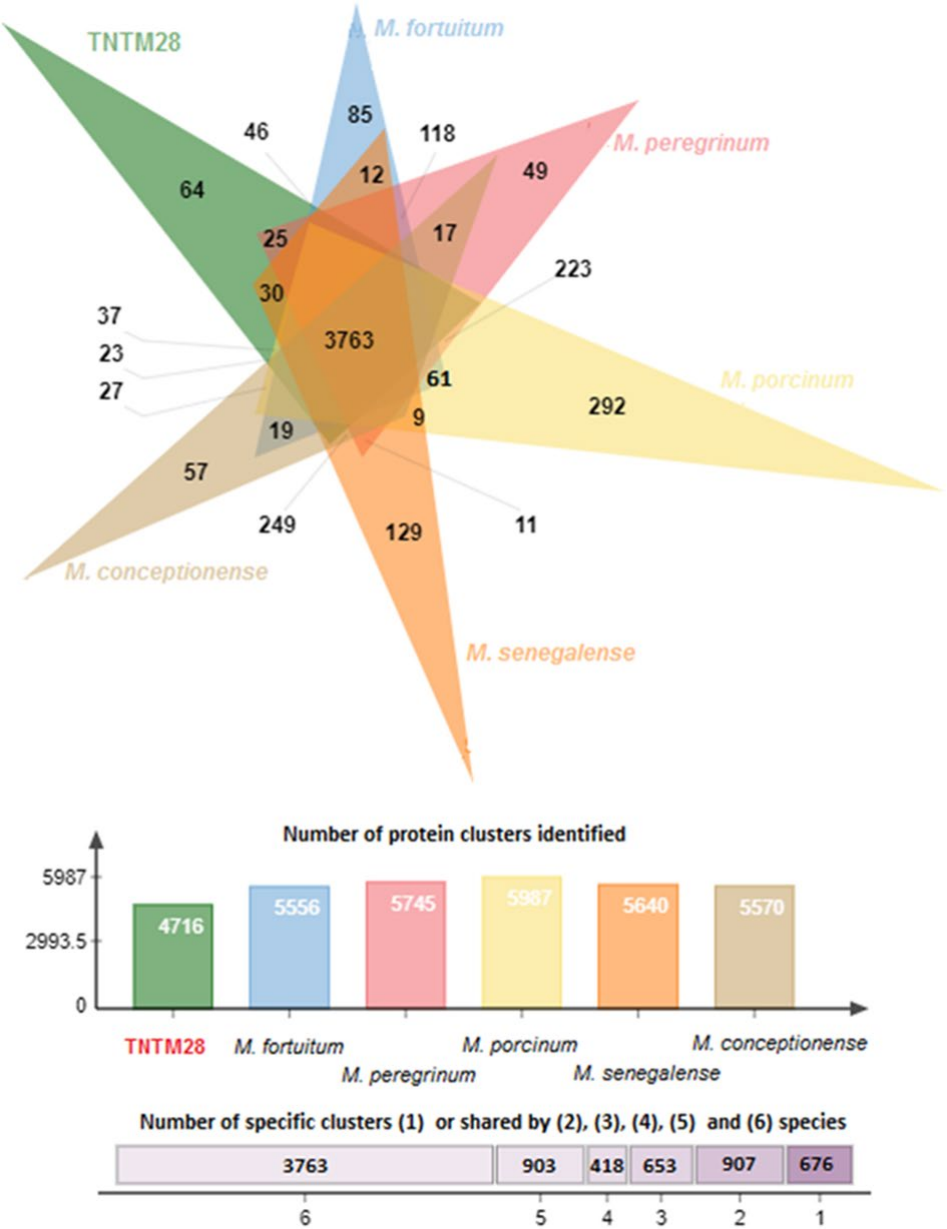
Venn diagram illustrating the distribution of shared and specific clusters of orthologous groups in TNTM28 and 5 *M. fortuitum* complex species.

The COGs content of the genome sequence of TNTM28 was analyzed and compared to that of five *M. fortuitum* complex species (*M. fortuitum, M. peregrinum, M. porcinum, M. senegalense, and M. conceptionense*). As shown in Fig. 3, the total number of genes in the six species varied from 4716 to 5570. Curiously, TNTM28 displayed the lowest number of genes, which reflects its particular phylogenetic status. Overall, 3763 shared COGs have been identified among the six species (Fig. 3). We found 64 genes unique to TNTM28, which could have contributed to specific phenotypic traits. These TNTM28-specific genes grouped into 18 gene ontology (GO) categories, particularly energy production and conversion (Supplementary Fig. S2).

The TNTM28 genome sequence was functionally annotated using the orthoMCL database, according to which 32% of the genes have been assigned to either ill-defined functional categories (“R” and “S” categories) or had no homologue (Table 2). The most represented functional categories were “lipid transport and metabolism” (category I; ~ 8.7%), “transcription” (category K; ~ 8.0%), “secondary metabolites biosynthesis, transport and catabolism” (category Q; ~ 7.9%), energy production and conversion (category C, ~ 6.8%), and “amino acid transport and metabolism (category E, ~ 6.0%).

**Table 2:** Number and proportion of genes associated with the 25 general COG functional categories.

Code	Value	% of total	Class description
[J]	162	3.1195840555	Translation, ribosomal structure and biogenisis
[A]	15	0.2888503755	RNA processing and modification
[K]	418	8.0492971308	Transcription
[L]	171	3.2928942808	Replication, recombination and repair
[B]	1	0.0192566917	Chromatin structure and dynamics
[D]	40	0.770267668	Cell cycle control, mitosis and meiosis
[Y]	0	0	Nuclear structure
[V]	50	0.962834585	Defense mechanisms
[T]	155	2.9847872136	Signal transduction mechanisms
[M]	163	3.1388407472	Cell wall/membrane biogenesis
[N]	13	0.2503369921	Cell motility
[Z]	0	0	Cytoskeleton
[W]	0	0	Extracellular structures
[U]	25	0.4814172925	Intracellular trafficking, secretion and vesicular transport
[O]	121	2.3300596957	Posttranslational modification, protein turn-over, chaperones
[C]	352	6.7783554785	Energy production and conversion
[G]	195	3.7550548816	Carbohydrate transport and metabolism
[E]	307	5.911804352	Amino acid transport and metabolism
[F]	77	1.4827652609	Nucleotide transport and metabolism
[H]	180	3.4662045061	Coenzyme transport and metabolism
[I]	450	8.6655112652	Lipid transport and metabolism
[P]	225	4.3327556326	Inorganic ion transport and metabolism
[Q]	413	7.9530136723	Secondary metabolites biosynthesis, transport and catabolism
[R]	669	12.8827267475	General function prediction only
[S]	371	7.1442326208	Function unknown
[–]	620	11.9391488542	Not in COGs

### Phylogenomic analysis

**Figure 4:**
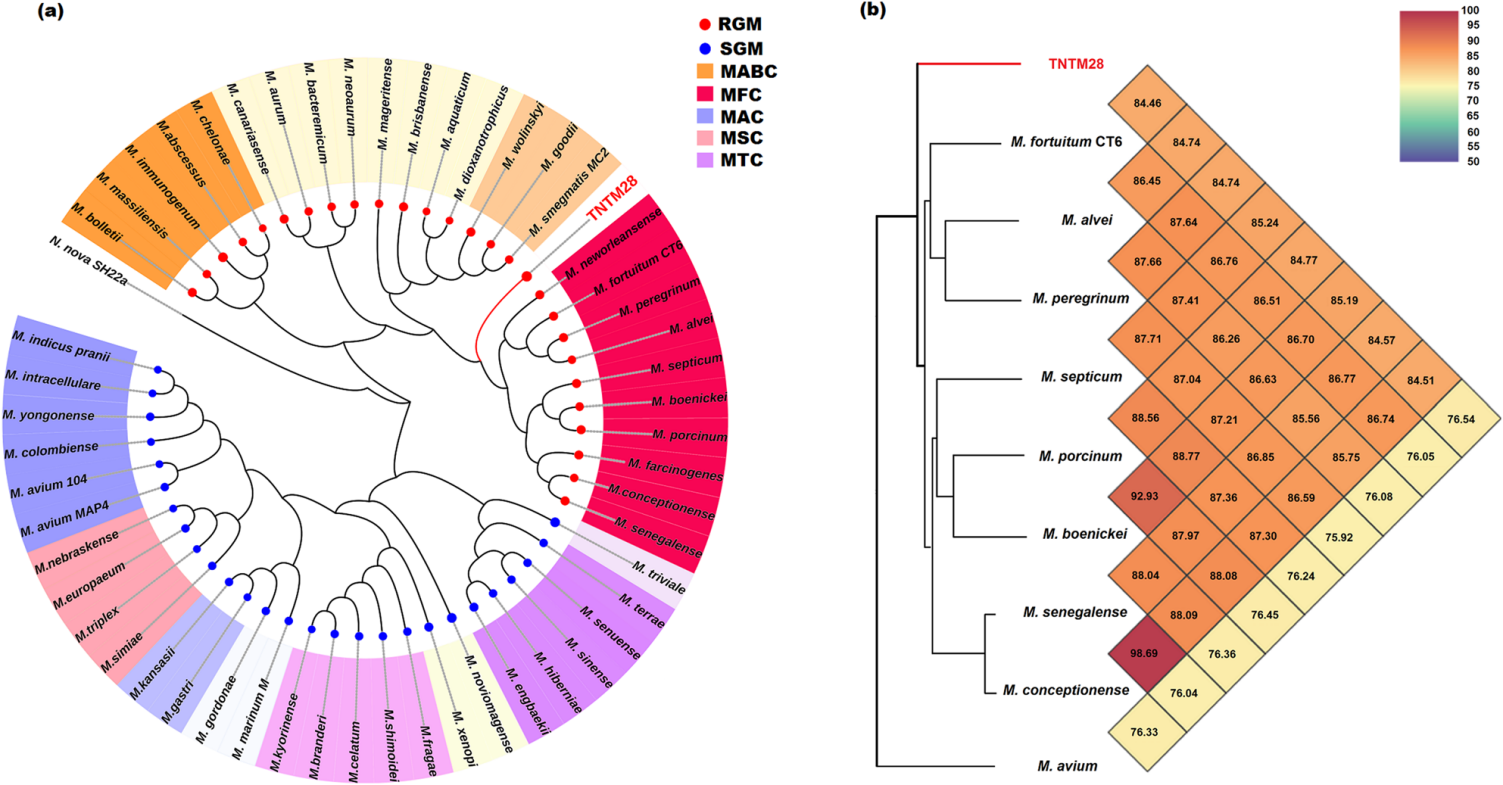
Phylogenomic of TNTM28. (**a**) Phylogenomic tree based on Mauve alignement identity matrix. (**b**) Heatmap generated with OrthoANI values of TNTM28 strain and other closest species of the *M*. *fortuitum* complex.

The phylogenetic placement of TNTM28 within the *Mycobacterium* genus was carried out based on MAUVE alignment of the genome sequence of 53 mycobacterial species. As shown in Fig. 4a, TNTM28 branched off from the common ancestor of the *M. fortuitum* complex, occupying a separate phylogenetic branch intermediate between this complex and *M. smegmatis*. Orthologous average nucleotide identity (orthoANI) scores rangin from 84.46 (*M. fortuitum* CT6) to 85.24% (*M. septicum*) were obtained (Fig. 4b).

### Genes potentially acquired through horizontal transfer

The identification of five prophage regions in the genome of TNTM28 is a witness of the occurrence of putative lateral gene transfer (LGT) events during its evolution. Therefore, we sought for horizontally transferred genomic islands (GIs). These acquired regions may encode several beneficial factors endowing the bacillus with a new virulence and drug resistance patterns, as well as higher adaptability to changing environmental conditions. Using the IslandViewer 4 platform and its improved GI prediction tool, the IslandPath-DIMOB^21^, eight GIs varying in length from 8510 to 37,995 bp have been identified (Supplementary Fig. S3, Supplementary Table S1). Overall, these horizontally transferred regions accounted for 2.28% of TNTM28 genome size and entailed 150 genes (2.88% of the coding capacity), 66% of which encoded hypothetical proteins.

Among the transferred genes with a known function, a large part was devoted to cell wall/membrane biogenesis and/or integrity (*n* = 7), transcription, as there were 6 helix-turn-helix (HTH) transcriptional regulators of various families (TetR/AcrR, GntR, and AraC), and detoxification (*n* = 4). We identified reductases (*n* = 2) and dehydrogenases (*n* = 2), which are likely to be involved in energy production and conversion, antitoxins (RelB and HipB), genes involved in lipid metabolism (hsaD and fgD), DNA repair (mutT2 and xseA), and carbohydrate metabolism. In addition, a copy of a hemagglutinin gene (hbhA) had been gained through a HGT event.

### Putative pathogenic features encoded in the TNTM28 genome

**Figure 5:**
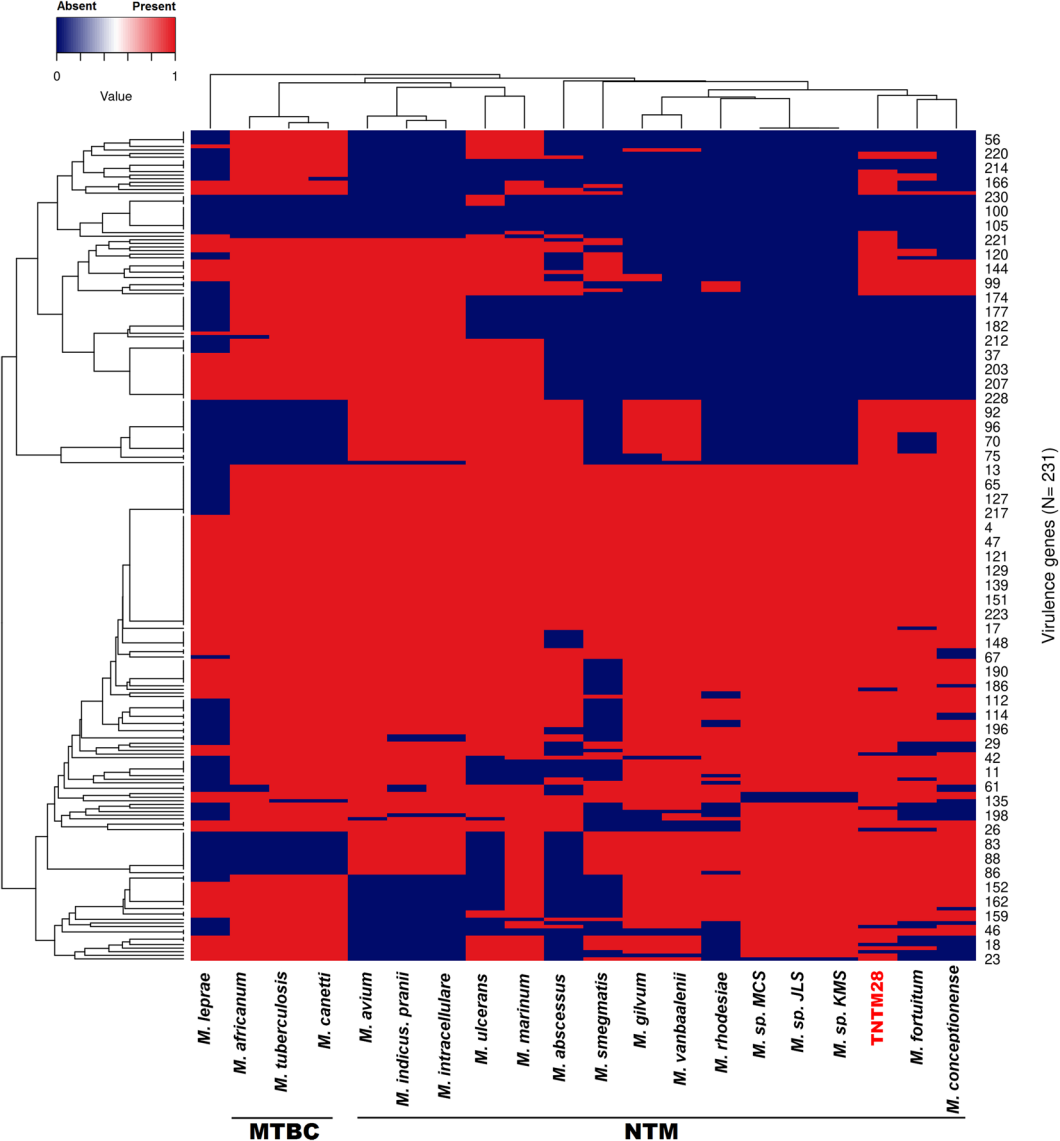
Heatmap showing the pathogenomic profile of TNTM28 compared with other mycobacterial species.

The genome of TNTM28 was found to contain a range of genes which in other mycobacteria are involved in mycobacterial pathogenicity, as defined within the virulence factor database^22^, endowing this new species with a relatively high probability (0.804) of being a pathogen for humans, as predicted by PathogenFinder^23^. A detailed distribution of 231 putative virulence genes among related SGM and pathogenic mycobacteria is provided in Supplementary Table S2. In addition, a heatmap was generated with dendograms showing the clustering of 20 mycobacterial species based on the presence or absence of each one of the 231 virulence genes, which further confirmed the relatedness of TNTM28 and the *M. fortuitum* group (Fig. 5).

Of particular interest, the TNTM28 genome contained an almost complete ESX-1 locus. As shown in Supplementary Fig. 4Sa, this type VII secretion locus, which in many SGM species is involved in virulence, lacked only espJ and espK, with all genes critical for secretion, namely *EccA* to *EccE* and *MycP,* being present. In pathogenic mycobacteria, including *Mycobacterium marinum* and *Mycobacteirum leprae*, ESX-1 secretion and function have been shown to be dependent upon the distal *espACD* operon, which has not been found in RGM genomes analysed so far^24^. Strikingly, scrutiny of the TNTM28 genome uncovered a distal operon comprising three genes (GEECPEIM_00946, GEECPEIM_00945, and GEECPEIM_00944) whose organization resembled that of the *espACD* operon (Supplementary Fig. 4Sb), with one gene, GEECPEIM_00945, showing significant orthology with the EspC product, as predicted by reciprocal best hits analysis (Supplementary data 1 to 3). The other two genes, GEECPEIM_00946 and GEECPEIM_00944, though positioned in a way similar to that of *espA* and *espD*, respectively, displayed no siginificant orthology with the latters. A similar situation was found in *M. fortuitum* (Supplementary Fig. 4Sb), but neither in *M. abscessus* nor in *M. smegmatis* (data not shown).

Like other NTMs, the TNTM28 genome harbored the ESX-3 locus. In addition, it contained an ESX-4 locus, representing the most ancestral ESX system known for mycobacteria^25,26^. However, the ESX-4 locus of TNTM28 contained no orthologue of *eccE*_*4*_, which in *M. abscessus* was deemed crucial for ESX-4 functions, notably with regard to the ability to block phagosomal acidification^27^.

Furthermore, the genome of TNTM28 encoded a large number (*n* = 40) of *mce* proteins, a gene family shown to be critical for invasion and persistence of mycobacteria in host macrophages and non-phagocytic mammalian cells, with *mce4* being implicated in cholesterol catabolism^28,29^. Homologs to *M. tuberculosis mce1A, mce1B*, *mce3E*, *mce3F*, which are lacking in *M. abscessus*, have been found in the TNTM28 genome. Importantly, the latter genome contained the full set of *mce4* genes (mce4A to mce4F). Additional *mce* genes (mce5 to mce9) specified by other NTM species and *Actinomycetales* species have been found^30,31^.

Like the majority of RGM species, we identified a few members of the PE/PPE multigene families, mainly those associated with the three Esx clusters (Esx-1, Esx3, and Esx-4)^31^.

We also identified seven members of the Sec export system (secA, secD, secE, secF, secG, secY, and yajC), which have been shown to be critical for *M. tuberculosis* virulence as they ensure the transport to the cytoplasmic membrane, and beyond, of several virulence factors. In particular, the presence of secA and secY, the motor protein and the major component of the translocon, respectively, may have endowed TNTM28 with the ability to ensure many functions important for its survival in the host^32^. Besides, we identified at least three sec-independent protein secretion pathway components.

Survival of mycobacteria within the host is greatly dependent upon their ability to produce cell wall-associated lipids, siderophores and other biologically active molecules^33,34^. In this respect, the genome of TNTM28 was found to be well equipped since it contained 40 genes involved in polyketide biosynthesis and 17 others involved in non-ribosomal peptide synthesis (NRPS). The existence of a *pks15* gene copy along with *ppsA-E* genes is noteworthy. In slow growing mycobacteria this locus was shown to be involved in the synthesis of phenolic glycolipids (PGL), representing major virulence factors of pathogenic mycobacteria^35^, whose presence in TNTM28 can now be experimentally verified. Furthermore, TNTM28 genome contained a gene copy of *mmpL8,* which encodes a product required for the synthesis of sulfolipid-1 (SL-1), a compound that is able to prevent the fusion of phagosome with lysosome to form the phagolysosome in macrophages. It also blocks oxidative phosphorylation and inhibits the production of reactive oxygen^36^. Yet, unlike *M. abscessus*, the TNTM28 genome lacked genes encoding phospholipase C-type enzymes, whose function in certain SGM has previously been proposed as virulence factor^37^, a hypothesis that has recently been dismissed^38^.

### Assessment of TNTM28 intramacrophagic growth

**Figure 6:**
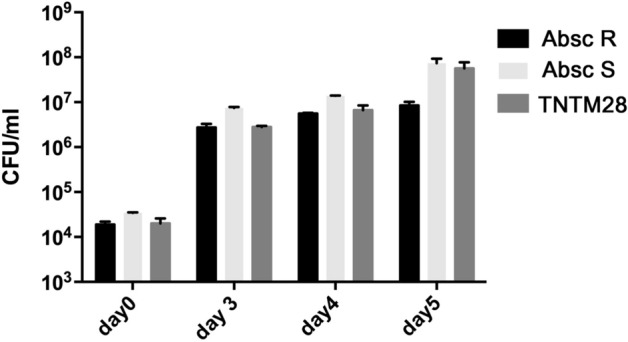
Growth of TNTM28 in THP-1 human macrophages as compared to smooth and rough *M. abscessus* variants (Absc S and Absc R, respectively). The number of CFU was determined at the indicated times post-infection. Error bars indicate the s.e.m., based on the results from 3 independent experiments.

The particular phenotypic and genomic features of TNTM28, which all converged towards its pathogenicity have promted us to assess its ability to replicate in macrophages. As shown in Fig. 6, TNTM28 replicated efficiently in PMA-differentiated THP-1 macrophages, with titers comparable to smooth and rough variants of *M. abscessus*.

## Discussion

In the present study we described the genome sequence of TNTM28, a new rapidly growing NTM species. Several features might indicate that this species could be endowed with a virulence potential. Firstly, it has been recovered from a patient presenting with a typical pulmonary disease. Review of the patient's clinical record by taking into account the American Thoracic Society (ATS)/Infectious Disease Society of America (IDSA) guidelines, argued for a true NTM pulmonary disease^39^. Secondly, TNTM28 displayed two main phenotypic features reminiscent of pathogenic mycobacteria. Indeed, TNTM28 colonies showed a rough morphotype, and Ziehl–Neelsen-stained cells tended to form large aggregates. Both characteristics have been associated with persistence inside phagocytes, which is most likely linked to the bacillus’s ability to escape from phagocytosis^5,39^. Another argument for an enhanced level of pathogenicity in TNTM28 was its ability to replicate in macrophages equally well as *M. abscessus*, one of the most pathogenic RGM species^40,41^.

Phylogenetic and phylogenomic analyses linked TNTM28 to the *M. fortuitum* complex. This finding came as no surprise given the ubiquitous presence of *M. fortuitum*-related species in the environment. Furthermore, *M. fortuitum* encompasses a large group of emerging opportunistic pathogens whose members have frequently been associated with pathogenic conditions in both immunocompetent and immunocompromised individuals^11^. Of particular interest, this new species, which has derived immediately from the common ancestor of all members of the *M. fortuitum* complex, displayed a smaller genome size compared to other pathogenic RGM (~ 1 Mb difference), such as *M. fortuitum* and *M. abscessus* complexes. Such a genomic reduction could have tightened the parasitic lifestyle of TNTM28, thereby enhancing its pathogenicity^42^.

As detailed in the Results section, the genome of TNTM28 contained a range of potential virulence genes, which might have promoted its ability for intracellular replication and persistence. Indeed, compared to other RGMs, TNTM28 specified numerous genes whose homologues exist essentially in pathogenic mycobacteria and are thus worthy of consideration. Among these was the *mgtC* gene, a key player in intramacrophage survival, being important for virulence in diverse intracellular pathogens^43,44^. This gene is usually absent from the genomes of several RGM, such as *M. fortuitum*, *M. smegmatis*, *M. gilvum*, etc., but like TNTM28, this gene does exist in the genome of *M. abscessus*. This finding is consistent with the fact that the latter two species were found to replicate equally well in macrophages. Furthermore, the genome of TNTM28 harbored a copy of the *clgR gene*, which has been shown *in M. tuberculosis* to be involved in the modulation of phagosome maturation^45^, and which was lacking in the majority of RGM, with the exception of *M. smegmatis*. In *M. tuberculosis*, ClgR activates the transcription of genes encoding a larger network of protein homeostatic and regulatory systems. ClgR-regulated transcriptional activation of these systems is essential for *M. tuberculosis* to replicate in macrophages by enabling the bacillus to control the phagosome pH^45^. By contrast, TNTM28 lacked the *sapM* gene, which encodes a secretory acid phosphatase, and whose disruption in *M. tuberculosis* translated into the inability of the mutant to arrest the phagosomal maturation with a severe growth defect in THP-1 macrophages^46^. Among other genes of importance was *trpD*, which encodes an anthranilate phosphoribosyltransferase involved in tryptophan biosynthesis. In SGM, *trpD* has been shown to play important roles during infection^47^. Aside from TNTM28, only *M. abscessus* was found to contain a gene copy of *trpD*. Interestingly, this gene proved essential for lung colonization by *M. tuberculosis* in mice^48^. Another distinctive feature of TNTM28, yet shared with *M. fortuitum* only, was the existence of a *treS* gene, which encodes a trehalose synthase enzyme, converting trehalose into maltose and vice versa. Deletion of the *treS* gene was shown to significantly prolonge the time to death in a chronic infection model in mice^49^.

Additional genes allowing TNTM28 to cope with the harsh intramacrophagic environment appear to have been brought by HGT. At least seven genes (murB, lprQ, fhaB, yidC, yidD, fgD2, and ispH1) involved in cell wall biogenesis might have been transferred to TNTM28, as such. These genes are of particular interest given the critical role played by the mycobacterial cell wall, whose extraordinarily complex nature significantly contribute to the ability of pathogenic mycobacteria to manipulate and evade human immune system^50,51^. Furthermore, and considering the vital role of cholesterol for optimal growth and persistence within the host^52–54^, the transfer of *hsaD*, a gene encoding a hydrolase involved in cholesterol catabolism, is likely to be of significance for TNTM28 virulence. Indeed, HsaD proved essential for survival of *M. tuberculosis* inside macrophages^55^. Moreover, and with regard to the cholesterol metabolism, it is worth mentioning that TNTM28 genome contained nearly the full set of *mce* genes, including the mce4 complex, which has been shown to be essential for cholesterol import^56,57^.

Among other notable putatively transferred genes that might have increased TNTM28 resistance to macrophage defensive arsenals was *mutT2*, given its pivotal role in protecting the bacillus against reactive oxygen species^58^.

Compared to the majority of RGM, TNTM28 harbored a near-complete ESX-1 locus, which could have endowed it with a relatively enhanced virulence, although the role of ESX-1 in RGM species such as *M. smegmatis* is rather linked to horizontal gene transfer than to virulence^59^. The ESX-1 region, along with ESX-3 and ESX-4 are ancestral regions and were thus found in the genomes of most mycobacteria^60^. The role of ESX-1 in survival in the macrophage and the overall bacillus’s pathogenicity has been largely demonstrated for MTBC members and slow growing mycobacteria, whose genome encode a complete ESX-1 system, as well as an associated *espACD* operon, which was not found in RGM so far^61–63^. It is worth noting that we have identified in TNTM28, but neither in *M. abscessus* nor in *M. smegmatis*, a locus structurally similar to *espACD*, and which, most intriguingly, was found to encode for an ortholog of EspC, the main modulator of ESX-1 function^24^. Therefore, it remains to be seen whether such an operon, does serve the same function(s) as the *espACD* operon of pathogenic mycobacteria. Should it be the case, this may also compensate for the lack in TNTM28 of the gene encoding Eis N-acetyl transferase protein, whose deletion mutant in *M. abscessus* proved strongly attenuated in macrophages^64^. Moreover, TNTM28 contained an ESX-4 copy, which in the absence of ESX-1, was shown to play a prominent role in *M. abscessus* growth in vivo^27^.

In summary, we identified a new RGM species displaying several phenotypic and genomic hallmarks that argue for its pathogenicity. Acquisition of TNTM28 virulence traits seemed to have benefited of a highly permissive environment for gene exchange, thereby favoring transition to pahogenicity. Because TNTM28 deep rooting in the phylogenetic tree, compared to the *Mycobacterium fortuitum* complex, being the unique representative of a newly derived branch, we propose a new taxonomic entity with the provisional name “*Mycobacterium fortunisiensis* sp. Nov”, which also refers to Tunis, the origin of isolation.

## Methods

### De novo sequencing and assembly

Genomic DNA (gDNA) was extracted using standard phenol-choloroform method and was sequenced on the HiSeq2500 Technology (Illumina Inc., San Diego, CA, USA) with paired-end application. The gDNA was quantified using Quant-iT™ PicoGreen^®^ ds DNA reagent, (Invitrogen, CA, USA). Paired-end library was constructed using "NEXTflex PCRFree Kit" according to Nextflex Illumina protocol. Automated cluster generation and paired end sequencing with dual index reads were performed in a single run in 2 × 107-pb. The 5.469.922 paired-end reads were firstly processed with FastQC and Trimmomatic sotwares^65^ before de novo genome assembling.

Paired-end reads were assembled using SPAdes genome assembler v.3.10.1^66^. Illumina reads were re-mapped into the scaffolds using the paired-end mode of Bwa mem v0.7.4^67^ with default parameters. After converting output SAM files to BAM files by SAMtools^68^, coverage mapping was computed by BEDtools v2.17.0^69^.

Filtered Scaffolds were ordered and oriented using CONTIGuator 2.7.4^70^ using *M. fortuitum* CT6, complete genome (Genbank CP011269) as reference in order to distinguish chromosome scaffolds and unplaced scaffolds. We used progressiveMauve for multiple genome alignment^71^.

### Genome annotation and phylogenetic analyses

Functionnal annotation was performed using the Prokaryotic Genome Annotation System (Prokka) v1.12 pipeline^72^. CRISPR loci were searched for using the CRISPRfinder program online^73^ (last update, 2017-05-09). The genomic circular representation of TNTM28 was generated using CGView server (http://stothard.afns.ualberta.ca/cgview_server/) and putative prophages were found using PHASTER (PhAge Search Tool) software^74^ based on the actinobacteriophage Database at phageDB.org and the online plasmid search tool http://plasmid.med.havard.edu/PLASMID/home.xhtml.

The prediction of tRNA was processed using ARAGORN program^75^, whereas ribosomal RNAs were predicted using RNAmmer^76^.

We performed functional annotation of genes using the Clusters of Orthologous Groups (COGs) database (http://www.ncbi.nlm.nih.gov/COG) using BLASTP (E value < 1e−5 and > 50% coverage).

The Phylogenomic tree was constructed using an identity matrix based on Mauve software (http://gel.ahabs.wisc.edu/mauve) genome alignment^71^. OrthoANI values were calculated between TNTM28 and 9 sequenced *Mycobacterium* species genomes using Orthologous Average Nucleotide Identity Tool^77^. Putative virulence genes were found using Pathogen Finder tool^23^ against the VFDB database (http://www.mgc.ac.cn/VFs/).

Horizontally transferred Genomic Islands (GIs) were identified using IslandPahth-DIMOB^78^.

Hierarchical clustering of closely related sets of virulence genes was generated after z-score normalization of the data using Euclidean distance.

### Pan genome analysis

The pan genome analysis, the core accessory, and unique genes was performed using the Bacterial Pan Genome Analysis Tool (BPGA)^79^.

ANI and DDH values were calculated using the GGDC version 2.0 online tool^80^.

### In vitro growth assessment of TNTM28 in THP-1 derived macrophages

THP-1 human monocyte-like cells (TIB-202D) were purchased from the American type culture collection (ATCC), directly amplified and stored in liquid nitrogen. Only low passage cells (number of passages < 11) were used in the experiments. The purchased THP-1 cell line was authenticated and tested against microbial contaminants, including mycoplasmas, by ATCC.

THP-1 cells were grown in RPMI 1640, GlutaMAX (Life Technologies) containing 10% heat-inactivated fetal bovine serum (Life Technologies), seeded at a density of 7.5 × 10^4^ cells per well in 96-well plates and differentiated into macrophages by incubation with 50 mM phorbol-myristate-acetate (PMA) for 3 days.

For infection, bacteria cultured in Sauton medium without agitation were sonicated, added to macrophages at a multiplicity of infection (MOI) of 0.05 (~ 1 bacteria per 20 THP cells), and incubated for 2 h. Sauton medium was used because it allows the production of more complex polar lipids^81^. After phagocytosis, 0.1 mg/ml of amikacin was added for one hour to eliminate extracellular bacteria and cells were incubated for up to 6 days at 37 °C and 5% CO_2_. At various times, the macrophages were lysed with 0.1% Triton-X100 in PBS and the lysates were plated in serial dilutions on 7H11 + OADC plates to determine the intracellular survival of the bacteria in c.f.u. The experiments were performed at least four biological replicas, each in triplicate (technical replicas).

## Supplementary Information

Supplementary Information.

## Data Availability

The *rpoB*, *hsp65*, 16 S rRNA and *sodA* gene sequences of strain TNTM28 was deposited in GenBank under accession number MK762879, MK751438, MK630280 and MK778075, respectively. Illumina reads for TNTM28 have been deposited at GenBank under accession number VOMB00000000. All data generated and analysed during this study are included in this manuscript and its supplementary information files.
