# Supplementary material for: Phenotypic and genomic hallmarks of a novel, potentially pathogenic rapidly growing Mycobacterium species related to the Mycobacterium fortuitum complex
Source: Sci Rep. 2021 Jun 21;11:13011. doi: 10.1038/s41598-021-91737-8 (PMC8217490; doi:10.1038/s41598-021-91737-8)

Phenotypic and genomic hallmarks of a novel, potentially pathogenic rapidly growing *Mycobacterium* species related to the *Mycobacterium fortuitum* complex

Reem Gharbi[1], Varun Khanna[2], Wafa Frigui[3], Besma Mhenni[1], Roland Brosch[3], Helmi Mardassi[1*]

[1] Unit of Typing & Genetics of Mycobacteria, Laboratory of Molecular Microbiology, Vaccinology, and Biotechnology Development, Institut Pasteur de Tunis, Université de Tunis El Manar;

[2] Institut Pasteur, Hub Bioinformatique et Biostatistique, C3BI, Unité de Services et de Recherche, USR 3756, Institut Pasteur CNRS, Paris, France;

[3] Institut Pasteur (IP), Unit for Integrated Mycobacterial Pathogenomics, 75015 Paris, France.

*** Corresponding author**

E. mail: helmi.merdassi@pasteur.rns.tn (HM)
Tel: +216 71 845 368

Supplementary table S1. Coordinates and gene content of TNTM28 genomic islands.

Island start	Island end	Length	Method	Gene ID	Locus	Gene start	Gene end	Strand	Product
789634	804212	14578	Predicted by at least one method		BLECMCFO_00736	789634	790338	-1	putative HTH-type transcriptional regulator
789634	804212	14578	Predicted by at least one method		BLECMCFO_00737	790445	791788	1	hypothetical protein
789634	804212	14578	Predicted by at least one method		BLECMCFO_00738	791878	792273	1	putative HTH-type transcriptional regulator
789634	804212	14578	Predicted by at least one method	hbhA	BLECMCFO_00739	792335	793003	1	Heparin-binding hemagglutinin
789634	804212	14578	Predicted by at least one method		BLECMCFO_00740	793073	793357	1	hypothetical protein
789634	804212	14578	Predicted by at least one method		BLECMCFO_00741	793362	793769	1	hypothetical protein
789634	804212	14578	Predicted by at least one method	deoC	BLECMCFO_00742	793769	794446	1	Deoxyribose-phosphate aldolase
789634	804212	14578	Predicted by at least one method		BLECMCFO_00743	794456	795262	-1	hypothetical protein

789634	804212	14578	Predicted by at least one method		BLECMCFO_00744	795370	796185	-1	Hydrolase
789634	804212	14578	Predicted by at least one method		BLECMCFO_00745	796239	796958	-1	hypothetical protein
789634	804212	14578	Predicted by at least one method		BLECMCFO_00746	797082	797570	-1	hypothetical protein
789634	804212	14578	Predicted by at least one method	murB	BLECMCFO_00747	797615	798718	1	UDP-N-acetylenolpyruvoylglucosamine reductase
789634	804212	14578	Predicted by at least one method	lprQ	BLECMCFO_00748	798725	800053	1	L,D-transpeptidase 5
789634	804212	14578	Predicted by at least one method		BLECMCFO_00749	800578	801495	1	hypothetical protein
789634	804212	14578	Predicted by at least one method		BLECMCFO_00750	801492	801776	1	hypothetical protein
789634	804212	14578	Predicted by at least one method	fhaA	BLECMCFO_00752	802147	803613	1	FHA domain-containing protein FhaA
789634	804212	14578	Predicted by at least one method	fhaB	BLECMCFO_00753	803745	804212	1	FHA domain-containing protein FhaB

828610	837120	8510	Predicted by at least one method	dnaA	BLECMCFO_00776	828610	830121	-1	Chromosomal replication initiator protein DnaA
828610	837120	8510	Predicted by at least one method	rpmH	BLECMCFO_00777	830634	830777	1	50S ribosomal protein L34
828610	837120	8510	Predicted by at least one method	rnpA	BLECMCFO_00778	830829	831164	1	Ribonuclease P protein component
828610	837120	8510	Predicted by at least one method	yidD	BLECMCFO_00779	831161	831517	1	Putative membrane protein insertion efficiency factor
828610	837120	8510	Predicted by at least one method	yidC	BLECMCFO_00780	831510	832580	1	Membrane protein insertase YidC
828610	837120	8510	Predicted by at least one method		BLECMCFO_00781	832620	833162	1	hypothetical protein
828610	837120	8510	Predicted by at least one method	rsmG	BLECMCFO_00782	833236	833952	1	Ribosomal RNA small subunit methyltransferase G
828610	837120	8510	Predicted by at least one method	soj_1	BLECMCFO_00783	833955	834941	1	Sporulation initiation inhibitor protein Soj

828610	837120	8510	Predicted by at least one method	parB	BLECMCFO_00784	834996	836036	1	putative chromosome-partitioning protein ParB
828610	837120	8510	Predicted by at least one method		BLECMCFO_00785	836269	837120	1	hypothetical protein
1181822	1203006	21184	Predicted by at least one method	glbN	BLECMCFO_01061	1181822	1182187	1	Group 1 truncated hemoglobin GlbN
1181822	1203006	21184	Predicted by at least one method		BLECMCFO_01066	1182776	1183945	-1	Putative prophage phiRv2 integrase
1181822	1203006	21184	Predicted by at least one method		BLECMCFO_01067	1183945	1184181	-1	hypothetical protein
1181822	1203006	21184	Predicted by at least one method		BLECMCFO_01068	1184186	1184506	-1	hypothetical protein
1181822	1203006	21184	Predicted by at least one method		BLECMCFO_01069	1184499	1185821	-1	hypothetical protein
1181822	1203006	21184	Predicted by at least one method		BLECMCFO_01070	1185818	1186237	-1	hypothetical protein
1181822	1203006	21184	Predicted by at least one method	yvoA_1	BLECMCFO_01071	1186338	1187117	-1	HTH-type transcriptional repressor YvoA

1181822	1203006	21184	Predicted by at least one method	mutT2_1	BLECMCFO_01072	1187210	1187638	1	Putative 8-oxo-dGTP diphosphatase 2
1181822	1203006	21184	Predicted by at least one method		BLECMCFO_01073	1187650	1187853	-1	hypothetical protein
1181822	1203006	21184	Predicted by at least one method		BLECMCFO_01074	1187920	1192881	-1	hypothetical protein
1181822	1203006	21184	Predicted by at least one method		BLECMCFO_01075	1193904	1195232	-1	hypothetical protein
1181822	1203006	21184	Predicted by at least one method		BLECMCFO_01076	1195339	1195641	1	hypothetical protein
1181822	1203006	21184	Predicted by at least one method		BLECMCFO_01077	1195642	1195941	1	hypothetical protein
1181822	1203006	21184	Predicted by at least one method		BLECMCFO_01078	1195957	1196430	-1	hypothetical protein
1181822	1203006	21184	Predicted by at least one method		BLECMCFO_01079	1196440	1197981	-1	hypothetical protein
1181822	1203006	21184	Predicted by at least one method		BLECMCFO_01080	1198453	1198725	1	hypothetical protein
1181822	1203006	21184	Predicted by at least one		BLECMCFO_01081	1198832	1199140	-1	hypothetical protein

			method						
1181822	1203006	21184	Predicted by at least one method		BLECMCFO_01082	1199232	1199462	-1	hypothetical protein
1181822	1203006	21184	Predicted by at least one method		BLECMCFO_01083	1199522	1200157	1	hypothetical protein
1181822	1203006	21184	Predicted by at least one method		BLECMCFO_01084	1200157	1200543	1	hypothetical protein
1181822	1203006	21184	Predicted by at least one method		BLECMCFO_01085	1200747	1201094	-1	hypothetical protein
1181822	1203006	21184	Predicted by at least one method		BLECMCFO_01086	1201148	1201639	1	hypothetical protein
1181822	1203006	21184	Predicted by at least one method		BLECMCFO_01087	1201742	1201906	-1	hypothetical protein
1181822	1203006	21184	Predicted by at least one method		BLECMCFO_01088	1202596	1203006	1	hypothetical protein
1181822	1203006	21184	Predicted by at least one method		BLECMCFO_01089	1203003	1203761	-1	L,D-transpeptidase 4
2176615	2185615	9000	Predicted by at least one method		BLECMCFO_02035	2175539	2176618	-1	hypothetical protein

2176615	2185615	9000	Predicted by at least one method	crcB	BLECMCFO_02036	2176615	2176980	-1	Putative fluoride ion transporter CrcB 1
2176615	2185615	9000	Predicted by at least one method	crcB1	BLECMCFO_02037	2176977	2177375	-1	Putative fluoride ion transporter CrcB 1
2176615	2185615	9000	Predicted by at least one method	pgm	BLECMCFO_02038	2177447	2179093	1	Phosphoglucomutase
2176615	2185615	9000	Predicted by at least one method	pbuE	BLECMCFO_02039	2179090	2180331	1	Purine efflux pump PbuE
2176615	2185615	9000	Predicted by at least one method		BLECMCFO_02041	2181504	2182922	1	hypothetical protein
2176615	2185615	9000	Predicted by at least one method		BLECMCFO_02042	2183190	2184827	-1	hypothetical protein
2176615	2185615	9000	Predicted by at least one method		BLECMCFO_02043	2185268	2185615	1	hypothetical protein
2490764	2500321	9557	Predicted by at least one method		BLECMCFO_02336	2490764	2491924	1	Lactate 2-monooxygenase
2490764	2500321	9557	Predicted by at least one method		BLECMCFO_02337	2491934	2493445	-1	hypothetical protein
2490764	2500321	9557	Predicted by at least one		BLECMCFO_02338	2493452	2493856	-1	hypothetical protein

			method						
2490764	2500321	9557	Predicted by at least one method	xerC_2	BLECMCFO_02339	2494023	2494919	1	Tyrosine recombinase XerC
2490764	2500321	9557	Predicted by at least one method	hsaD_3	BLECMCFO_02340	2494927	2495826	1	4,5:9,10-diseco-3-hydroxy-5,9, 17-trioxoandrosta-1(10),2-diene-4-oate hydrolase
2490764	2500321	9557	Predicted by at least one method		BLECMCFO_02341	2495807	2496316	-1	hypothetical protein
2490764	2500321	9557	Predicted by at least one method	rpsB	BLECMCFO_02342	2496615	2497451	1	30S ribosomal protein S2
2490764	2500321	9557	Predicted by at least one method	tsf	BLECMCFO_02343	2497472	2498299	1	Elongation factor Ts
2490764	2500321	9557	Predicted by at least one method		BLECMCFO_02344	2498437	2499276	-1	hypothetical protein
2490764	2500321	9557	Predicted by at least one method	fgd2_3	BLECMCFO_02345	2499467	2500321	1	F420-dependent hydroxymycolic acid dehydrogenase

4489570	4506435	16865	Predicted by at least one method	dhaAF	BLECMCFO_04230	4489570	4490481	-1	Haloalkane dehalogenase
4489570	4506435	16865	Predicted by at least one method		BLECMCFO_04231	4490478	4491251	-1	hypothetical protein
4489570	4506435	16865	Predicted by at least one method		BLECMCFO_04232	4491248	4491922	-1	hypothetical protein
4489570	4506435	16865	Predicted by at least one method		BLECMCFO_04233	4492004	4492570	-1	hypothetical protein
4489570	4506435	16865	Predicted by at least one method		BLECMCFO_04234	4492715	4493587	1	2-(acetamidomethylene)succinate hydrolase
4489570	4506435	16865	Predicted by at least one method		BLECMCFO_04235	4493598	4493765	-1	hypothetical protein
4489570	4506435	16865	Predicted by at least one method		BLECMCFO_04236	4493779	4494060	-1	hypothetical protein
4489570	4506435	16865	Predicted by at least one method		BLECMCFO_04237	4494689	4496113	1	hypothetical protein
4489570	4506435	16865	Predicted by at least one method		BLECMCFO_04238	4496199	4496879	1	hypothetical protein

4489570	4506435	16865	Predicted by at least one method		BLECMCFO_04239	4496887	4497402	-1	hypothetical protein
4489570	4506435	16865	Predicted by at least one method	calB_3	BLECMCFO_04240	4497609	4499039	1	Coniferyl aldehyde dehydrogenase
4489570	4506435	16865	Predicted by at least one method		BLECMCFO_04241	4499051	4499452	-1	hypothetical protein
4489570	4506435	16865	Predicted by at least one method	relB	BLECMCFO_04242	4499743	4500156	-1	Antitoxin RelB
4489570	4506435	16865	Predicted by at least one method		BLECMCFO_04243	4500157	4500555	-1	hypothetical protein
4489570	4506435	16865	Predicted by at least one method		BLECMCFO_04244	4500664	4500918	-1	hypothetical protein
4489570	4506435	16865	Predicted by at least one method		BLECMCFO_04245	4501104	4501475	-1	Ribonuclease VapC32
4489570	4506435	16865	Predicted by at least one method	ychF	BLECMCFO_04246	4502423	4503496	-1	Ribosome-binding ATPase YchF
4489570	4506435	16865	Predicted by at least one method		BLECMCFO_04247	4503668	4504765	1	hypothetical protein

4489570	4506435	16865	Predicted by at least one method	ispH1	BLECMCFO_04248	4504784	4505779	-1	4-hydroxy-3-methylbut-2-enyl diphosphate reductase 1
4489570	4506435	16865	Predicted by at least one method		BLECMCFO_04249	4505851	4506435	1	hypothetical protein
4489570	4506435	16865	Predicted by at least one method	xseA	BLECMCFO_04250	4506432	4507661	1	Exodeoxyribonuclease 7 large subunit
5090696	5128691	37995	Predicted by at least one method		BLECMCFO_04837	5090696	5090956	-1	hypothetical protein
5090696	5128691	37995	Predicted by at least one method		BLECMCFO_04838	5091604	5094084	1	hypothetical protein
5090696	5128691	37995	Predicted by at least one method		BLECMCFO_04839	5094385	5095272	-1	hypothetical protein
5090696	5128691	37995	Predicted by at least one method		BLECMCFO_04840	5096342	5096569	-1	hypothetical protein
5090696	5128691	37995	Predicted by at least one method		BLECMCFO_04841	5096849	5097100	1	hypothetical protein
5090696	5128691	37995	Predicted by at least one method		BLECMCFO_04842	5097341	5097646	1	hypothetical protein

5090696	5128691	37995	Predicted by at least one method		BLECMCFO_04843	5097646	5097933	1	hypothetical protein
5090696	5128691	37995	Predicted by at least one method		BLECMCFO_04844	5097930	5099366	1	hypothetical protein
5090696	5128691	37995	Predicted by at least one method		BLECMCFO_04845	5099363	5099848	1	hypothetical protein
5090696	5128691	37995	Predicted by at least one method		BLECMCFO_04846	5100630	5100788	1	hypothetical protein
5090696	5128691	37995	Predicted by at least one method		BLECMCFO_04847	5100785	5101648	-1	hypothetical protein
5090696	5128691	37995	Predicted by at least one method	hipB	BLECMCFO_04848	5102345	5102662	-1	Antitoxin HipB
5090696	5128691	37995	Predicted by at least one method		BLECMCFO_04849	5102714	5103511	1	hypothetical protein
5090696	5128691	37995	Predicted by at least one method		BLECMCFO_04850	5104081	5104836	1	hypothetical protein
5090696	5128691	37995	Predicted by at least one method		BLECMCFO_04851	5104849	5105967	-1	hypothetical protein
5090696	5128691	37995	Predicted by at least one method		BLECMCFO_04852	5105997	5107163	-1	hypothetical protein

5090696	5128691	37995	Predicted by at least one method		BLECMCFO_04853	5107160	5108368	-1	hypothetical protein
5090696	5128691	37995	Predicted by at least one method		BLECMCFO_04854	5108698	5109249	-1	hypothetical protein
5090696	5128691	37995	Predicted by at least one method		BLECMCFO_04855	5109242	5111776	-1	hypothetical protein
5090696	5128691	37995	Predicted by at least one method	xerD_3	BLECMCFO_04856	5111776	5112330	-1	Tyrosine recombinase XerD
5090696	5128691	37995	Predicted by at least one method		BLECMCFO_04857	5113073	5113216	-1	hypothetical protein
5090696	5128691	37995	Predicted by at least one method		BLECMCFO_04858	5113244	5113813	-1	hypothetical protein
5090696	5128691	37995	Predicted by at least one method		BLECMCFO_04859	5113810	5114595	-1	hypothetical protein
5090696	5128691	37995	Predicted by at least one method		BLECMCFO_04860	5114592	5114768	-1	Putative conjugal transfer protein
5090696	5128691	37995	Predicted by at least one method		BLECMCFO_04861	5114901	5115653	1	hypothetical protein
5090696	5128691	37995	Predicted by at least one		BLECMCFO_04862	5115715	5116959	1	hypothetical protein

			method						
5090696	5128691	37995	Predicted by at least one method	xerC_3	BLECMCFO_04863	5116976	5118178	1	Tyrosine recombinase XerC
5090696	5128691	37995	Predicted by at least one method	xerC_4	BLECMCFO_04864	5118165	5120396	1	Tyrosine recombinase XerC
5090696	5128691	37995	Predicted by at least one method		BLECMCFO_04865	5120393	5120803	1	hypothetical protein
5090696	5128691	37995	Predicted by at least one method		BLECMCFO_04866	5120800	5121459	-1	putative HTH-type transcriptional regulator
5090696	5128691	37995	Predicted by at least one method		BLECMCFO_04867	5121603	5122115	1	hypothetical protein
5090696	5128691	37995	Predicted by at least one method	kstR_4	BLECMCFO_04868	5122112	5122735	1	HTH-type transcriptional repressor KstR
5090696	5128691	37995	Predicted by at least one method		BLECMCFO_04869	5122809	5123234	1	hypothetical protein
5090696	5128691	37995	Predicted by at least one method		BLECMCFO_04870	5123359	5126445	1	Acyltrehalose exporter MmpL10

5090696	5128691	37995	Predicted by at least one method		BLECMCFO_04871	5126545	5127615	-1	Putative conjugal transfer protein
5090696	5128691	37995	Predicted by at least one method		BLECMCFO_04872	5127612	5128691	-1	hypothetical protein
5265344	5277948	12604	Predicted by at least one method		BLECMCFO_05031	5271986	5272264	-1	hypothetical protein
5265344	5277948	12604	Predicted by at least one method	virS	BLECMCFO_05032	5272399	5273409	1	HTH-type transcriptional regulator VirS
5265344	5277948	12604	Predicted by at least one method		BLECMCFO_05033	5273473	5273904	1	hypothetical protein
5265344	5277948	12604	Predicted by at least one method		BLECMCFO_05034	5274109	5274339	1	hypothetical protein
5265344	5277948	12604	Predicted by at least one method		BLECMCFO_05035	5274366	5274791	1	hypothetical protein
5265344	5277948	12604	Predicted by at least one method		BLECMCFO_05036	5274821	5275309	1	hypothetical protein
5265344	5277948	12604	Predicted by at least one method		BLECMCFO_05037	5275553	5275732	-1	hypothetical protein
5265344	5277948	12604	Predicted by at least one		BLECMCFO_05038	5275729	5275911	-1	hypothetical protein

			method						
5265344	5277948	12604	Predicted by at least one method	xerC_5	BLECMCFO_05039	5275908	5277167	-1	Tyrosine recombinase XerC
5265344	5277948	12604	Predicted by at least one method		BLECMCFO_05041	5277436	5277948	-1	hypothetical protein

Supplementary table S2. Presence (1) or absence (0) of 231 putative virulence genes in the genome of TNTM28 as compared to a representative set of rapidly and slowly growing mycobacteria.

Virulence genes	*M. abscessus*	TNTM28	*M. fortuitum*	*M. conceptionense*	*M. indicus pranii*	*M. avium*	*M. intracellulare*	*M. tuberculosis*	*M. africanum*	*M. canetti*	*M. leprae*	*M. ulcerans*	*M. marinum*	*M. gilvum*	*M. vanbaalenii*	*M. sp JLS*	*M. sp KMS*	*M. sp MCS*	*M. rhodesiae*	*M. smegmatis*
glnA1	1	1	1	1	1	1	1	1	1	1	1	1	1	1	1	1	1	1	1	1
leuD	1	1	1	1	1	1	1	1	1	1	1	1	1	1	1	1	1	1	1	1
lysA	1	1	1	1	1	1	1	1	1	1	1	1	1	1	1	1	1	1	1	1
proC	1	1	1	1	1	1	1	1	1	1	1	1	1	1	1	1	1	1	1	1
purC	1	1	1	1	1	1	1	1	1	1	1	1	1	1	1	1	1	1	1	1
trpD	1	1	0	0	0	0	0	0	0	0	1	1	0	0	0	0	0	0	0	0
narX	0	1	1	0	0	0	0	1	1	0	0	0	0	0	0	0	0	0	0	0
narG	0	1	1	1	1	1	1	1	1	1	0	0	0	1	1	1	1	1	1	0
narH	0	1	1	1	1	1	1	1	1	1	0	0	0	1	1	1	1	1	1	0
narJ	0	1	1	1	1	1	1	1	1	1	0	0	0	1	1	1	1	1	1	0
narI	0	1	1	1	1	1	1	1	1	1	0	0	0	1	1	1	1	1	1	0
narK2	1	1	1	1	0	0	0	1	1	1	0	0	0	1	1	1	1	1	1	1
nuoG	1	1	1	1	1	1	1	1	1	1	0	1	1	1	1	1	1	1	1	1
erp	1	1	1	0	1	1	1	1	1	1	1	1	1	1	1	1	1	1	1	1
hbhA	1	1	1	1	1	1	1	1	1	1	1	1	1	1	1	1	1	1	1	1
mmA4	0	0	0	1	1	1	1	1	1	1	1	1	1	1	1	1	1	1	1	1
cmaA2	1	1	0	1	1	1	1	1	1	1	1	1	1	1	1	1	1	1	1	1
fadD26	0	0	0	0	0	0	0	1	1	1	1	1	1	1	1	1	1	1	0	1
fadD28	1	1	1	1	1	1	1	1	1	1	1	1	1	1	1	1	1	1	0	1
ppsA	0	1	0	0	0	0	0	1	1	1	1	1	1	1	1	1	1	1	0	1
ppsB	0	1	0	0	0	0	0	1	1	1	1	1	1	1	1	1	1	1	0	1
ppsC	0	1	0	0	0	0	0	1	1	1	1	1	1	0	0	1	1	1	0	0
ppsD	0	1	0	0	0	0	0	1	1	1	1	1	1	1	1	0	0	0	0	1
ppsE	0	1	1	0	0	0	0	1	1	1	1	1	1	1	1	1	1	1	0	1

papA5	0	0	0	0	0	0	0	1	1	1	1	1	1	1	1	1	1	1	0	0
mas	1	0	0	1	1	1	1	1	1	1	1	1	1	0	0	1	1	1	0	0
mmpL7	0	0	0	0	0	0	0	1	1	1	1	1	1	0	0	0	0	0	0	0
drrA	0	1	0	0	1	1	1	1	1	1	1	1	1	1	1	1	1	1	1	1
drrB	0	1	0	0	1	1	1	1	1	1	0	1	1	1	1	1	1	1	1	1
drrC	0	1	0	0	1	1	1	1	1	1	1	1	1	1	1	1	1	1	1	0
TreS	0	1	1	0	0	0	0	1	1	1	0	0	0	0	0	0	0	0	0	0
lppx	0	0	0	0	0	0	0	1	1	1	0	0	0	0	0	0	0	0	0	0
pks1	0	0	0	0	1	1	1	1	1	1	1	0	0	0	0	0	0	0	0	0
pks15	0	1	0	0	0	0	0	1	1	1	0	0	0	0	0	0	0	0	0	0
pks5	1	1	1	0	0	0	0	1	1	1	0	1	1	0	0	0	0	0	0	0
pks7	0	0	0	0	1	1	1	1	1	1	1	1	1	0	0	0	0	0	0	0
pks12	0	0	0	0	1	1	1	1	1	1	1	1	1	0	0	0	0	0	0	0
pks10	0	1	1	1	1	1	1	1	1	1	1	1	1	0	0	0	0	0	0	1
choD	1	1	1	1	1	1	1	1	1	1	1	1	1	0	0	0	0	0	0	1
hsaC	1	1	1	0	1	1	1	1	1	1	1	1	1	0	0	0	0	0	0	0
pcaA	1	1	1	1	1	1	1	1	1	1	1	1	1	1	1	1	1	1	1	1
stf0	1	1	1	1	1	1	1	1	1	1	0	0	1	0	0	1	1	1	1	1
papA2	1	1	1	1	1	1	1	1	1	1	0	0	0	1	1	1	1	1	0	1
papA1	0	1	1	1	0	0	0	1	1	1	0	0	0	0	0	1	1	1	0	0
pks2	1	1	1	1	1	1	1	1	1	1	1	1	1	0	0	1	1	1	0	0
mmpL8	0	1	1	1	0	0	0	1	1	1	0	0	0	0	0	1	1	1	0	0
kasB	1	1	1	1	1	1	1	1	1	1	1	1	1	1	1	1	1	1	1	1
icl	1	1	1	1	1	1	1	1	1	1	0	1	1	1	1	1	1	1	1	1
lipF	0	1	1	1	1	1	1	1	1	1	1	1	1	1	1	1	1	1	1	1
sapM	0	0	0	0	1	1	1	1	1	1	0	1	1	0	0	0	0	0	0	1
cyp125	0	1	1	1	1	1	1	1	1	1	1	1	1	0	0	0	0	0	0	1
panC	1	1	1	1	1	1	1	1	1	1	1	1	1	1	1	1	1	1	1	1
panD	1	1	1	1	1	1	1	1	1	1	1	1	1	1	1	1	1	1	1	1
plcA	0	0	0	0	0	0	0	1	1	1	0	1	1	0	0	0	0	0	0	0

plcB	0	0	0	0	0	0	0	1	1	1	0	1	1	0	0	0	0	0	0	0
plcC	0	0	0	0	0	0	0	1	1	1	0	1	1	0	0	0	0	0	0	0
plcD	0	0	0	0	0	0	0	1	1	1	0	1	1	0	0	0	0	0	0	0
mce1A	0	1	1	1	1	1	1	1	1	1	1	1	1	1	1	1	1	1	1	1
mce1B	0	1	1	1	1	1	1	1	1	1	1	1	1	1	1	1	1	1	1	1
mce3E	0	1	1	0	0	1	1	1	0	1	0	1	1	1	1	1	1	1	1	1
mce3F	0	1	1	0	0	1	1	1	0	1	0	1	1	1	1	1	1	1	1	1
mce4A	1	1	1	1	1	1	1	1	1	1	0	1	1	1	1	1	1	1	1	1
mce4B	1	1	1	1	1	1	1	1	1	1	0	1	1	1	1	1	1	1	1	1
mce4C	1	1	1	1	1	1	1	1	1	1	0	1	1	1	1	1	1	1	1	1
mce4D	1	1	1	1	1	1	1	1	1	1	0	1	1	1	1	1	1	1	1	1
mce4E	1	1	1	1	1	1	1	1	1	1	0	1	1	1	1	1	1	1	1	1
mce4F	1	1	1	0	1	1	1	1	1	1	0	1	1	1	1	1	1	1	1	1
mce5A	1	1	0	1	1	1	1	0	0	0	0	1	1	1	1	0	0	0	0	0
mce5B	1	1	0	1	1	1	1	0	0	0	0	1	1	1	1	0	0	0	0	0
mce5C	1	1	0	1	1	1	1	0	0	0	0	1	1	1	1	0	0	0	0	0
mce5D	1	1	0	1	1	1	1	0	0	0	0	1	1	1	1	0	0	0	0	0
mce5E	1	1	0	1	1	1	1	0	0	0	0	1	1	1	1	0	0	0	0	0
mce5F	1	1	0	1	1	1	1	0	0	0	0	1	1	1	1	0	0	0	0	0
mce6A	1	1	1	1	1	1	1	0	0	0	0	1	1	0	1	0	0	0	0	0
mce6B	1	1	1	1	1	1	1	0	0	0	0	1	1	0	1	0	0	0	0	0
mce6C	1	1	1	1	0	0	0	0	0	0	0	1	1	0	0	0	0	0	0	0
mce6D	1	1	1	1	1	1	1	0	0	0	0	1	1	1	1	0	0	0	0	0
mce6E	1	1	1	1	1	1	1	0	0	0	0	1	1	1	1	0	0	0	0	0
mce6F	1	1	1	1	1	1	1	0	0	0	0	1	1	1	1	0	0	0	0	0
mce7A	0	1	1	1	1	1	1	0	0	0	0	0	1	1	1	1	1	1	1	1
mce7B	0	1	1	1	1	1	1	0	0	0	0	0	1	1	1	1	1	1	1	1
mce7C	0	1	1	1	1	1	1	0	0	0	0	0	1	1	1	1	1	1	1	1
mce7D	0	1	1	1	1	1	1	0	0	0	0	0	1	1	1	1	1	1	1	1
mce7E	0	1	1	1	1	1	1	0	0	0	0	0	1	1	1	1	1	1	1	1

mce7F	0	1	1	1	1	1	1	0	0	0	0	0	1	1	1	1	1	1	1	1
mce8A	0	1	1	1	1	1	1	0	0	0	0	0	1	1	1	1	1	1	0	1
mce8B	0	1	1	1	1	1	1	0	0	0	0	0	1	1	1	1	1	1	1	1
mce8C	0	1	1	1	1	1	1	0	0	0	0	0	1	1	1	1	1	1	1	1
mce8D	0	1	1	1	1	1	1	0	0	0	0	0	1	1	1	1	1	1	1	1
mce8E	0	1	1	1	1	1	1	0	0	0	0	0	1	1	1	1	1	1	1	1
mce8F	0	1	1	1	1	1	1	0	0	0	0	0	1	1	1	1	1	1	1	1
mce9A	1	1	1	1	1	1	1	0	0	0	0	1	1	1	1	0	0	0	0	0
mce9B	1	1	1	1	1	1	1	0	0	0	0	1	1	1	1	0	0	0	0	0
mce9C	1	1	1	1	1	1	1	0	0	0	0	1	1	1	1	0	0	0	0	0
mce9D	1	1	1	1	1	1	1	0	0	0	0	1	1	1	1	0	0	0	0	0
mce9E	1	1	1	1	1	1	1	0	0	0	0	1	1	1	1	0	0	0	0	0
mce9F	1	1	1	1	1	1	1	0	0	0	0	1	1	1	1	0	0	0	0	0
irtA	1	1	1	1	1	1	1	1	1	1	0	1	1	0	0	0	0	0	1	0
irtB	1	1	1	1	1	1	1	1	1	1	0	1	1	0	0	0	0	0	1	0
fxuC	0	0	0	0	0	0	0	0	0	0	0	0	0	0	0	0	0	0	0	0
fxuA	0	0	0	0	0	0	0	0	0	0	0	0	0	0	0	0	0	0	0	0
fxuB	0	0	0	0	0	0	0	0	0	0	0	0	0	0	0	0	0	0	0	0
fxbA	0	0	0	0	0	0	0	0	0	0	0	0	0	0	0	0	0	0	0	0
exiT	0	0	0	0	0	0	0	0	0	0	0	0	0	0	0	0	0	0	0	0
fxbBC	0	0	0	0	0	0	0	0	0	0	0	0	0	0	0	0	0	0	0	0
fxuD	0	0	0	0	0	0	0	0	0	0	0	0	0	0	0	0	0	0	0	0
ideR	1	1	1	1	1	1	1	1	1	1	1	1	1	1	1	1	1	1	1	1
mgtC	1	1	0	0	1	1	1	1	1	1	0	1	1	0	0	1	1	1	0	0
mbtH	1	1	1	1	1	1	1	1	1	1	0	1	1	1	1	1	1	1	1	1
mbtG	1	1	1	0	1	1	1	1	1	1	0	1	1	1	1	1	1	1	1	0
mbtF	1	1	1	1	1	1	1	1	1	1	0	1	1	1	1	1	1	1	1	0
mbtE	1	1	1	1	1	1	1	1	1	1	0	1	1	1	1	1	1	1	1	0
mbtD	1	1	1	1	1	1	1	1	1	1	0	1	1	1	1	1	1	1	0	0
mbtC	1	1	1	0	1	1	1	1	1	1	0	1	1	1	1	1	1	1	1	0

mbtB	1	1	1	1	1	1	1	1	1	1	0	1	1	1	1	1	1	1	1	0
mbtA	1	1	1	1	1	1	1	1	1	1	0	1	1	1	1	1	1	1	1	0
mbtJ	1	1	1	1	1	1	1	1	1	1	0	1	1	0	0	0	0	0	1	1
mbtI	1	1	1	1	1	1	1	1	1	1	0	1	1	0	0	0	0	0	0	0
fadD33	0	1	1	1	1	1	1	1	1	1	0	0	0	1	1	1	1	1	0	0
fadE29	0	1	1	0	1	1	1	1	1	1	0	1	1	0	0	0	0	0	0	1
relA	1	1	1	1	1	1	1	1	1	1	1	1	1	1	1	1	1	1	1	1
DevR/dosR	1	1	1	1	0	1	0	1	1	1	0	1	1	1	1	1	1	1	1	0
devS	1	1	1	1	0	1	0	1	1	1	0	1	1	1	1	1	1	1	1	0
mprA	1	1	1	1	1	1	1	1	1	1	1	1	1	1	1	1	1	1	1	1
mprB	1	1	1	1	1	1	1	1	1	1	1	1	1	1	1	1	1	1	1	1
phoP	1	1	1	1	1	1	1	1	1	1	0	1	1	1	1	1	1	1	1	1
phoR	1	1	1	1	1	1	1	1	1	1	0	1	1	1	1	1	1	1	1	1
prrA	1	1	1	1	1	1	1	1	1	1	1	1	1	1	1	1	1	1	1	1
prrB	1	1	1	1	1	1	1	1	1	1	1	1	1	1	1	1	1	1	1	1
sigA1rpoV	1	1	1	0	1	1	1	1	1	1	1	1	1	1	1	1	1	1	1	1
sigE	1	1	1	1	1	1	1	1	1	1	1	1	1	1	1	1	1	1	1	1
sigF	1	1	1	1	1	1	1	1	1	1	0	1	1	1	1	1	1	1	1	1
sigH	1	1	1	1	1	1	1	1	1	1	0	1	1	1	1	1	1	1	1	1
sigM	1	1	1	1	1	1	1	1	1	1	0	1	1	1	1	1	1	1	1	1
whiB3	1	1	1	0	1	1	1	0	1	0	1	1	1	1	1	0	0	0	1	1
lpqH	1	1	1	1	1	1	1	1	1	1	1	1	1	1	1	1	1	1	1	1
hspX	1	1	0	0	1	0	1	1	1	1	0	0	1	0	1	1	1	1	1	0
fbpA	1	1	1	1	1	1	1	1	1	1	1	1	1	1	1	1	1	1	1	1
fbpB	1	1	1	1	1	1	1	1	1	1	1	1	1	1	1	1	1	1	1	1
fbpC	1	1	1	1	1	1	1	1	1	1	1	1	1	1	1	1	1	1	1	1
pstA	1	1	1	1	1	1	1	1	1	1	1	1	1	1	1	0	0	0	1	1
caeA	0	1	1	1	1	1	1	1	1	1	1	1	1	1	1	0	0	0	1	1
oppA	1	1	0	0	1	1	1	1	1	1	1	1	1	0	0	0	0	0	0	1
oppB	0	1	1	1	1	1	1	1	1	1	1	1	1	0	0	0	0	0	0	1

oppC	0	1	1	1	1	1	1	1	1	1	1	1	1	1	0	0	0	0	0	1
oppD	0	1	1	1	1	1	1	1	1	1	1	1	1	1	0	0	0	0	0	1
Eis	1	0	0	1	0	0	0	1	1	1	0	0	1	1	1	1	1	1	0	0
pknD	0	1	1	1	1	1	1	1	1	1	1	1	1	1	1	1	1	1	1	1
pknE	1	1	1	1	1	1	1	1	1	1	1	1	1	1	1	1	1	1	1	1
pknG	1	1	1	1	1	1	1	1	1	1	1	1	1	1	1	1	1	1	1	1
secA2	1	1	1	1	1	1	1	1	1	1	1	1	1	1	1	1	1	1	1	1
esxA	0	1	1	1	0	0	0	1	1	1	1	0	1	1	1	1	1	1	1	0
esxB	0	1	1	1	0	0	0	1	1	1	0	0	1	1	1	1	1	1	1	0
eccA1	0	1	1	1	0	0	0	1	1	1	1	0	1	1	1	1	1	1	1	0
eccB1	0	1	1	0	0	0	0	1	1	1	1	0	1	1	1	1	1	1	1	0
eccCa1	0	1	1	1	0	0	0	1	1	1	1	0	1	1	1	1	1	1	1	0
eccCb1	0	1	1	1	0	0	0	1	1	1	1	0	1	1	1	1	1	1	1	0
PE35	0	1	1	1	0	0	0	1	1	1	0	0	1	1	1	1	1	1	1	0
eccD1	0	1	1	1	0	0	0	1	1	1	1	1	1	1	1	1	1	1	1	0
espK	0	1	0	0	0	0	0	0	0	0	0	0	1	0	0	0	0	0	0	0
eccE1	0	1	1	1	0	0	0	1	1	1	1	0	1	1	1	1	1	1	1	0
mycP1	0	1	1	1	0	0	0	1	1	1	1	0	1	1	1	1	1	1	1	0
espG1	1	1	1	1	0	0	0	1	1	1	1	0	1	0	0	0	0	0	0	1
espH	0	1	0	0	0	0	0	1	1	1	1	0	1	0	0	0	0	0	0	1
espC/espF	1	1	0	0	0	0	0	1	1	1	1	0	1	0	0	0	0	0	0	0
espA	0	1	0	0	0	0	0	1	1	1	1	0	1	0	0	0	0	0	0	0
espB	0	1	0	0	0	0	0	1	1	1	0	0	1	1	1	1	1	1	0	0
PPE68	0	1	1	1	0	0	0	1	1	1	1	0	1	1	1	1	1	1	1	0
espI	1	1	1	1	1	1	1	1	1	1	1	1	1	1	1	1	1	1	1	1
espJ	0	0	0	0	0	0	0	1	1	1	0	1	1	1	1	0	0	0	0	0
espL	0	1	1	1	0	0	0	1	1	1	1	1	1	1	1	1	1	1	1	0
espR	1	1	1	1	1	1	1	1	1	1	1	1	1	1	1	1	1	1	1	1
eccA2	0	0	0	0	1	1	1	1	1	1	0	0	0	0	0	0	0	0	0	0
eccE2	0	0	0	0	1	1	1	1	1	1	0	0	0	0	0	0	0	0	0	0

mycP2	0	0	0	0	1	1	1	1	1	1	0	0	0	0	0	0	0	0	0	0
eccD2	0	0	0	0	1	1	1	1	1	1	0	0	0	0	0	0	0	0	0	0
espG2	0	0	0	0	1	1	1	1	1	1	0	0	0	0	0	0	0	0	0	0
esxC	0	0	0	0	1	1	1	1	0	1	0	0	0	0	0	0	0	0	0	0
esxD	0	0	0	0	1	1	1	1	1	1	0	0	0	0	0	0	0	0	0	0
PPE69	0	0	0	0	1	1	1	1	1	1	0	0	0	0	0	0	0	0	0	0
PE36	0	0	0	0	1	1	1	1	1	1	0	0	0	0	0	0	0	0	0	0
eccC2	0	0	0	0	1	1	1	1	1	1	0	0	0	0	0	0	0	0	0	0
eccB2	0	0	0	0	1	1	1	1	1	1	0	0	0	0	0	0	0	0	0	0
eccA3	1	1	1	1	1	1	1	1	1	1	1	1	1	1	1	1	1	1	1	0
eccB3	1	1	1	1	1	1	1	1	1	1	1	1	1	1	1	1	1	1	1	0
eccC3	1	1	1	0	1	1	1	1	1	1	1	1	1	1	1	1	1	1	1	0
PE5	1	0	1	1	1	1	1	1	1	1	1	1	1	1	1	1	1	1	1	0
PPE4	1	0	0	0	1	1	1	1	1	1	0	1	1	1	1	1	1	1	0	0
esxH	1	1	1	1	1	1	1	1	1	1	1	1	1	1	1	1	1	1	1	0
espG3	1	1	1	1	1	1	1	1	1	1	1	1	1	1	1	1	1	1	1	0
eccD3	1	1	1	1	1	1	1	1	1	1	1	1	1	1	1	1	1	1	1	0
mycP3	1	1	1	1	1	1	1	1	1	1	1	1	1	1	1	1	1	1	1	0
eccE3	1	1	1	1	1	1	1	1	1	1	1	1	1	1	1	1	1	1	0	0
esxG	1	1	1	1	1	1	1	1	1	1	1	1	1	1	1	1	1	1	1	0
esxT	0	1	1	1	1	1	1	1	1	1	0	1	1	1	1	1	1	1	1	0
esxU	0	1	1	1	1	1	1	1	1	1	0	1	1	1	1	1	1	1	1	0
eccC4	1	1	1	1	1	1	1	1	1	1	0	1	1	1	1	1	1	1	0	0
cccD4	1	1	0	0	0	1	0	1	1	1	0	1	1	0	1	1	1	1	0	0
mycP4	1	1	0	1	1	1	1	1	1	1	0	0	0	1	1	1	1	1	1	0
eccB4	1	1	0	0	1	1	1	1	1	1	0	1	1	1	1	1	1	1	0	0
eccA5	0	0	0	0	1	1	1	1	1	1	1	1	1	0	0	0	0	0	0	0
eccE5	0	0	0	0	1	1	1	1	1	1	1	1	1	0	0	0	0	0	0	0
mycP5	0	0	0	0	1	1	1	1	1	1	1	1	1	0	0	0	0	0	0	0
eccD5	0	0	0	0	1	1	1	1	1	1	1	1	1	0	0	0	0	0	0	0

esxN	0	0	0	0	1	1	1	1	1	1	1	1	1	0	0	0	0	0	0	0
esxM	0	0	0	0	1	1	1	1	1	1	1	1	1	0	0	0	0	0	0	0
eccCb5	0	0	0	0	1	1	1	1	1	1	1	1	1	0	0	0	0	0	0	0
eccCa5	0	0	0	0	1	1	1	1	1	1	1	1	1	0	0	0	0	0	0	0
eccB5	0	0	0	0	1	1	1	1	1	1	1	1	1	0	0	0	0	0	0	0
PPE41	0	0	0	0	0	0	0	1	1	1	0	0	0	0	0	0	0	0	0	0
PPE25	0	0	0	0	1	1	1	1	1	1	0	1	1	0	0	0	0	0	0	0
PE18	0	0	0	0	1	1	1	1	1	1	0	1	1	0	0	0	0	0	0	0
PPE26	0	0	0	0	1	1	1	1	1	1	0	1	1	0	0	0	0	0	0	0
PPE27	0	0	0	0	0	0	0	1	1	1	0	0	0	0	0	0	0	0	0	0
PE19	0	0	0	0	1	1	1	1	1	1	0	1	1	0	0	0	0	0	0	0
ahpC	1	1	0	0	1	1	1	1	1	1	1	1	1	0	0	0	0	0	0	0
katG	1	1	1	1	1	1	1	1	1	1	0	1	1	1	1	1	1	1	1	1
sodC	1	1	1	1	1	1	1	1	1	1	1	1	1	1	1	1	1	1	1	1
sodA	1	1	1	1	1	1	1	1	1	1	1	1	1	0	0	1	1	1	0	0
VirS	0	1	1	0	0	0	0	1	1	1	0	1	1	0	0	0	0	0	0	0
clgR	0	1	0	0	1	1	1	1	1	1	1	1	1	0	0	0	0	0	0	1
rip1	0	1	1	1	1	1	1	1	1	1	1	1	1	1	1	1	1	1	1	1
SenX 3	1	1	1	1	1	1	1	1	1	1	1	1	1	1	1	1	1	1	1	1
RegX3	1	1	1	1	1	1	1	1	1	1	1	1	1	1	1	1	1	1	1	1
hspR	1	1	1	1	1	1	1	1	1	1	1	1	1	1	1	1	1	1	1	1
modA	1	1	1	1	1	1	1	1	1	1	1	1	1	1	1	1	1	1	1	1
fadD22	0	0	0	0	1	1	1	1	1	1	1	1	1	0	0	0	0	0	0	0
fadD29	0	0	0	0	1	1	1	1	1	1	1	1	1	0	0	0	0	0	0	0
mlsA1	0	0	0	0	0	0	0	0	0	0	0	1	0	0	0	0	0	0	0	0
mlsA2	0	0	0	0	0	0	0	0	0	0	0	1	0	0	0	0	0	0	0	0
mlsB	0	0	0	0	0	0	0	0	0	0	0	1	0	0	0	0	0	0	0	0

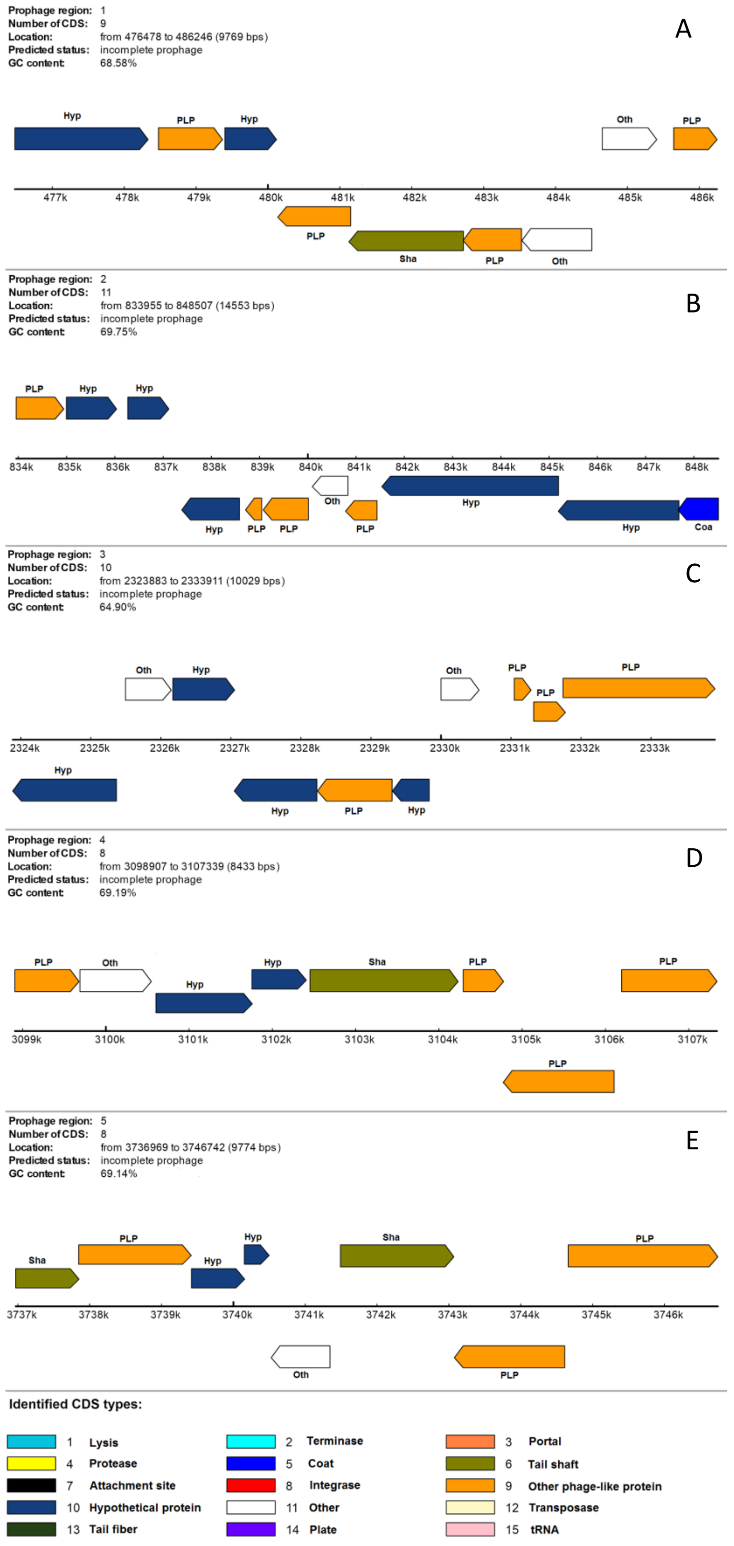

Supplementary figure S1. The five incomplete prophage regions (A to E) identified in the genome of TNTM28.

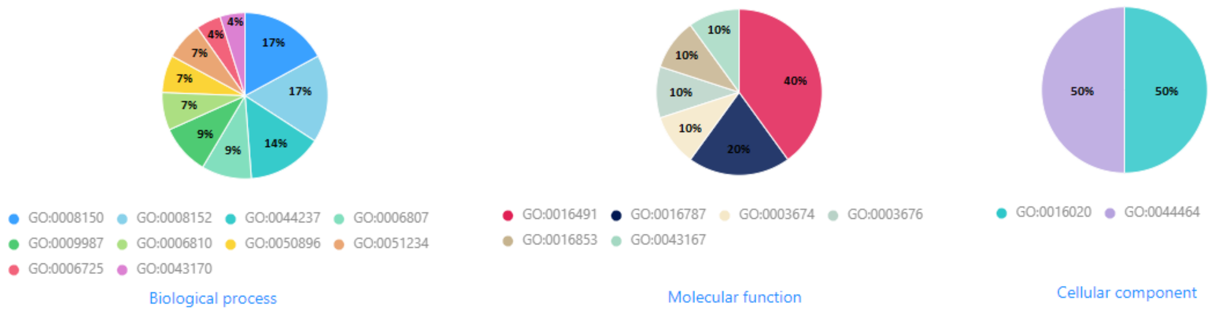

**Biological process**		
**Slimmed_GO**	**Count_of_unique_input_accessions**	**Name**
GO:0008150	7	biological_process
GO:0008152	7	metabolic process
GO:0044237	6	cellular metabolic process
GO:0006807	4	nitrogen compound metabolic process
GO:0009987	4	cellular process
GO:0006810	3	transport
GO:0050896	3	response to stimulus
GO:0051234	3	establishment of localization
GO:0006725	2	cellular aromatic compound metabolic process
GO:0043170	2	macromolecule metabolic process
**Molecular function**		
GO:0016491	3	oxidoreductase activity
GO:0016787	2	hydrolase activity
GO:0003674	1	molecular_function
GO:0003676	1	nucleic acid binding
GO:0016853	1	isomerase activity
GO:0043167	1	ion binding
GO:0016491	1	oxidoreductase activity
**Cellular component**		
GO:0016020	3	membrane
GO:0044464	3	cell part

Supplementary Figure S2. Distribution of TNTM28-specific genes into gene ontology (GO) categories.

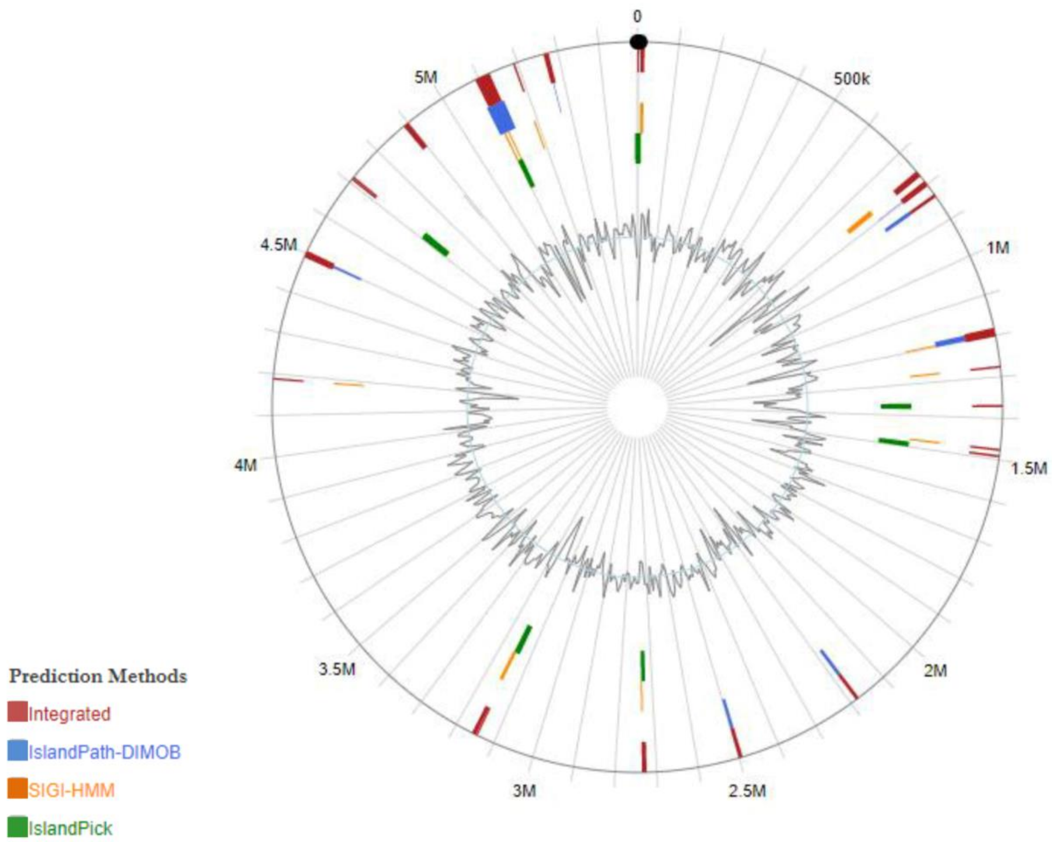

Supplementary Figure S3. Genomic islands identified in the TNTM28 genome using IslandViewer 4 platform and its improved GI prediction tool, the IslandPath-DIMOB.

(a)

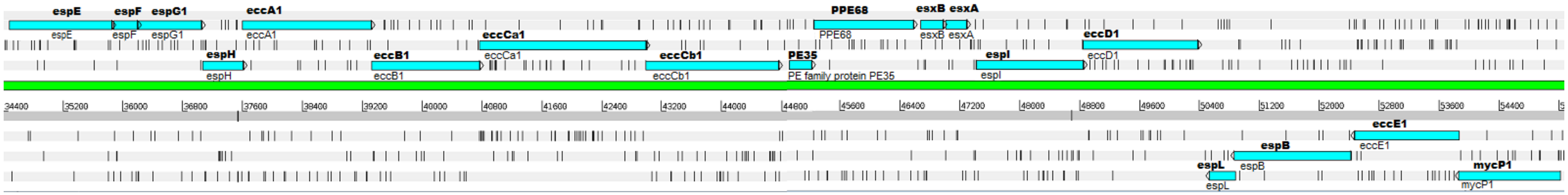

(b)

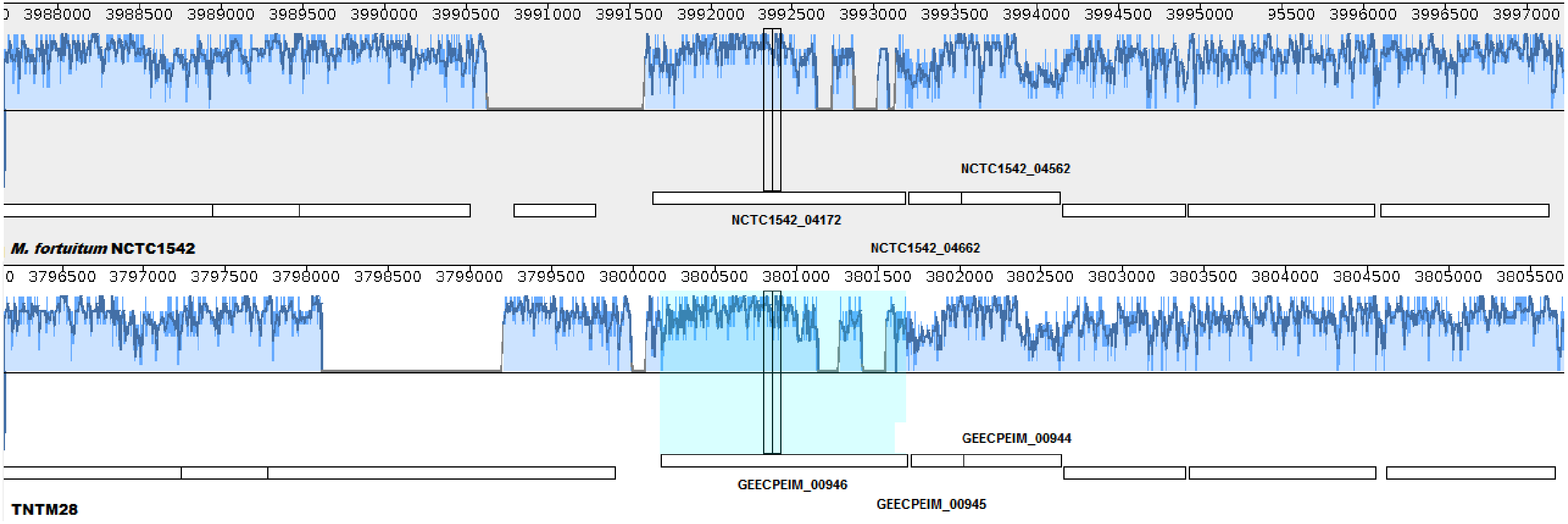

Supplementary Figure S4. (a) Genomic map of TNTM28 ESX-1 locus and **(b)** the distal operon found in both TNTM28 and *M. fortuitum*, which encodes an ortholog of EspC (GEECPEIM_00945 and NCTC1542_04662).

Supplementary data 1

Ortholog_RBH method_orthoMCL		Confidence Levels						
Species	Gene/Protein	Overall confidence Set weights	Orthology prediction	Orthology databases	Sequence alignment	Domain architecture	Functional motifs	Gene Ontology
**TNTM28**	**GEECPEIM_00946**	(1.5)	25.0%	0.0%	12.5%	0.0%	80.0%	N/A

Query	Ortholog	Global alignment					Local alignment				
		Length [aa]	Identity	Similarity	Gaps	Score	Length [aa]	Identity	Similarity	Gaps	Score
**P9WJE1_espA**	**GEECPEIM_00946**	817	12.5%	18.8%	60.0%	152.0	432	23.1%	35.0%	25.9%	155.0

Hypothetical protein

Graphical view   Domain alignment

**Query**	1		392
**GEECPEIM_00946**	1		377

Supplementary data 2

Ortholog-RBH method_orthoMCL		Confidence Levels						
Species	Gene/Protein	Overall confidence Set weights	Orthology prediction	Orthology databases	Sequence alignment	Domain architecture	Functional motifs	Gene Ontology
**TNTM28**	**GEECPEIM_00945**	(3.1)	50.0%	0.0%	24.1%	100.0%	75.0%	N/A

Query	Ortholog	Global alignment					Local alignment				
		Length [aa]	Identity	Similarity	Gaps	Score	Length [aa]	Identity	Similarity	Gaps	Score

Query	Ortholog	Global alignment					Local alignment				
		Length [aa]	Identity	Similarity	Gaps	Score	Length [aa]	Identity	Similarity	Gaps	Score
**P9WJD7**_espC	**GEECPEIM_00945**	108	24.1%	38.9%	10.2%	80.0	85	29.4%	45.9%	0.0%	94.0

Graphical view Domain alignment

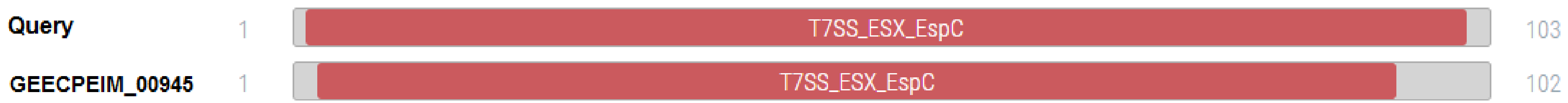

Supplementary data 3

Ortholog_RBH method_orthoMCL		Confidence Levels							
Species	Gene/Protein	Overall confidence Set weights	Orthology prediction	Orthology databases	Sequence alignment	Domain architecture	Functional motifs	Gene Ontology	PubMed papers
**TNTM28**	**GEECPEIM_00944**<br>**Hypothetical protein**	(2.4)	50.0%	0.0%	16.6%	0.0%	100.0%	N/A	N/A

Query	Ortholog	Global alignment					Local alignment				
		Length [aa]	Identity	Similarity	Gaps	Score	Length [aa]	Identity	Similarity	Gaps	Score

Query	Ortholog	Global alignment					Local alignment				
		Length [aa]	Identity	Similarity	Gaps	Score	Length [aa]	Identity	Similarity	Gaps	Score
**P9WJD5**_espD	**GEECPEIM_00944**	193	16.6%	26.9%	46.6%	76.5	73	31.5%	50.7%	11.0%	81.5

Graphical view   Domain alignment

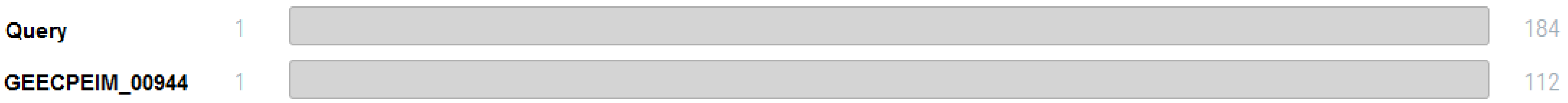

Supplement: Supplementary file 1 — Supplementary Information. [file 41598_2021_91737_MOESM1_ESM.pdf]
